# Interferon-γ signaling is associated with *BRCA1* loss-of-function mutations in high grade serous ovarian cancer

**DOI:** 10.1038/s41698-019-0103-4

**Published:** 2019-12-06

**Authors:** Horacio Cardenas, Guanglong Jiang, Jessica Thomes Pepin, J. Brandon Parker, Salvatore Condello, Kenneth P. Nephew, Harikrishna Nakshatri, Debabrata Chakravarti, Yunlong Liu, Daniela Matei

**Affiliations:** 1 Department of Obstetrics and Gynecology, Northwestern University, Chicago, IL USA; 2 Department of Medical and Molecular Genetics, Indiana University, Indianapolis, IN USA; 3 Department of BioHealth Informatics, Indiana University-Purdue University Indianapolis, Indianapolis, IN USA; 4 Department of Obstetrics and Gynecology, Indiana University, Indianapolis, IN USA; 5 Melvin and Bren Simon Cancer Center, Indianapolis, IN USA; 6 Medical Sciences, Indiana University School of Medicine, Bloomington, IN USA; 7 Department of Cellular and Integrative Physiology, Indiana University School of Medicine, Indianapolis, IN USA; 8 Departments of Surgery, Biochemistry and Molecular Biology, Indiana University, Indianapolis, IN USA; 9 Robert H Lurie Comprehensive Cancer Center, Chicago, IL USA; 10 Jesse Brown VA Medical Center, Chicago, IL USA

**Keywords:** Ovarian cancer, Epigenetics

## Abstract

Loss-of-function mutations of the breast cancer type 1 susceptibility protein (BRCA1) are associated with breast (BC) and ovarian cancer (OC). To identify gene signatures regulated by epigenetic mechanisms in OC cells carrying BRCA1 mutations, we assessed cellular responses to epigenome modifiers and performed genome-wide RNA- and chromatin immunoprecipitation-sequencing in isogenic OC cells UWB1.289 (carrying a BRCA1 mutation, BRCA1-null) and UWB1.289 transduced with wild-type BRCA1 (BRCA1+). Increased sensitivity to histone deacetylase inhibitors (HDACi) was observed in BRCA1-null vs. BRCA1+ cells. Gene expression profiles of BRCA1-null vs. BRCA1+ cells and treated with HDACi were integrated with chromatin mapping of histone H3 lysine 9 or 27 acetylation. Gene networks activated in BRCA1-null vs. BRCA1 + OC cells related to cellular movement, cellular development, cellular growth and proliferation, and activated upstream regulators included *TGFβ1, TNF*, and *IFN-γ*. The IFN-γ pathway was altered by HDACi in BRCA1+ vs. BRCA1-null cells, and in BRCA1-mutated/or low vs. BRCA1-normal OC tumors profiled in the TCGA. Key IFN-γ-induced genes upregulated at baseline in BRCA1-null vs. BRCA1+OC and BC cells included *CXCL10, CXCL11*, and *IFI16*. Increased localization of STAT1 in the promoters of these genes occurred in BRCA1-null OC cells, resulting in diminished responses to IFN-γ or to STAT1 knockdown. The IFN-γ signature was associated with improved survival among OC patients profiled in the TCGA. In all, our results support that changes affecting IFN-γ responses are associated with inactivating BRCA1 mutations in OC. This signature may contribute to altered responses to anti-tumor immunity in BRCA1-mutated cells or tumors.

## Introduction

Genetic or epigenetic inactivation of several tumor suppressor genes (TSGs), including *TP53*, *PTEN*, *BRCA1*, *BRCA2*, *DOK2*, *RB1*, and others^1–3^, are strongly associated with tumor initiation and progression of ovarian and breast cancers. The most commonly mutated TSG associated with hereditary OC, BRCA1, has been implicated in the initiation of events leading to transformation of the ovarian/fallopian tube epithelium.^4,5^ Inactivating BRCA1 mutations occur in approximately 5–10% of all high-grade serous ovarian cancers (HGSOC) and account for more than half of the inherited syndromes associated with OC.^6^ Additionally, epigenetic silencing of BRCA1 through promoter CpG island methylation occurs in approximately 20% of HGSOC,^1,7^ and has been linked to OC progression. The BRCA1 protein has four domains: the zinc-finger C3HC4 RING domain involved in protein ubiquitination, the BRCA1 serine (SCD) domain which contains phosphorylation sites responsible for localization of the protein to DNA damage sites, and two C-terminal (BRCT) domains that regulate DNA repair responses.^8,9^ Through its interaction with partner proteins, BRCA1 is part of the complex that repairs DNA double-strand breaks (DSB) and maintains the integrity of the genome.^10,11^ Tumor-associated BRCA1 mutations commonly affecting the BRCT domain confer increased sensitivity to DNA damaging agents and to poly ADP ribose polymerase (PARP) inhibitors and are associated with substantial transcriptional rewiring in cancer cells and tumors.^12–15^

Acetylation of histone H3 lysine residues at positions 9- and 27-marks active promoters and enhancers and is regulated by the concerted activities of histone acetyl transferases (HATs) and histone deacetylases (HDACs).^16^ Recent reports indicated that treatment with HDAC inhibitors of ovarian and breast cancer cells induce “BRCA-ness” features associated with increased response to DNA damage and PARP inhibitors.^17,18^ It had also been reported that the BRCT domain of BRCA1 interacts with histone deacetylases (HDAC) 1 and 2 and with other components of the chromatin remodeling complex^19^, supporting a potential association between presence of BRCA1 mutations and reorganization of histone marks delineating active regions of chromatin. Therefore, we sought to determine the transcriptomic signatures induced by HDAC inhibitors in BRCA1 mutated or in BRCA1-wild-type OC cells and their association with genome-wide distribution of H3 acetylation marks, thus defining differences in transcription-ready chromatin regions between cells harboring BRCA1 mutations versus wild type.

Here we compared the transcriptome of OC cells carrying a loss of function BRCA1 mutation and cells in which BRCA1 was restored at baseline and in response to treatment with an HDAC inhibitor. These analyses integrated with gene expression profiles of BRCA1-deficient (mutated BRCA1 or expression levels below the tenth quantile) or BRCA1-normal (not mutated BRCA1 and expression level above the first quartile) HGSOC tumors profiled in The Cancer Genome Atlas (TCGA) project^1^ pointed to an IFN-γ signature being hyper-active at baseline in BRCA1-mutated cancer cells. IFN-γ is secreted by natural killer, helper, and cytotoxic T lymphocytes and is involved in innate and adaptive immunity. Cellular signaling induced by the cytokine involves binding to its receptors (IFNGR1 and 2) and activation of the Janus kinase JAK–STAT pathway. Upon phosphorylation of Ser-727, STAT1 dimerizes and its homodimers translocate to the nucleus, localize to target gene promoters inducing transcription.^20^ IFN-γ has been recognized as a key player in anti-tumor immune responses.^21^ The cytokine induces cancer cell apoptosis and growth arrest^22–24^ and its secretion by activated CD8 cytotoxic T cells in the tumor milieu is responsible for T cell-induced anti-tumor effects. We showed that the IFN-γ signature in BRCA1-mutated cells and tumors carries prognostic significance in HGSOC, is associated with distinct patterns of H3K9 and H3K27 acetylation, and is functionally linked to partial resistance to IFN-γ stimulation. Our findings suggest a potential mechanism by which loss of function BRCA1 mutations could alter the response of cancer cells to external stimuli.

## Results

### Differential response to HDAC inhibitors based on the presence of a mutated BRCA1

Based on recent reports suggesting synergy between epigenome-targeting agents and PARP inhibitors,^25^ with HDAC inhibitors inducing a BRCA-ness state,^17,26^ we tested differential growth responses of isogenic cancer cells carrying or not a BRCA1 mutation to epigenome-modifying agents. We utilized the isogenic cells UWB1.289 and UWB1.289 transduced with BRCA1 (ref. ^27^) (hereafter referred to as BRCA1-null and BRCA1+, per ATCC nomenclature, respectively). The UWB1.289 cells carry a 2594delC mutation which introduces a stop at codon 845 leading to a truncated protein lacking a large portion of the carboxyl terminus. The deleted portion includes the SCD and the two BRCT domains. Consistent with a previous report,^27^ non-mutated *BRCA1 mRNA* and protein levels were very low, and H2AX phosphorylation (γH2AX) in response to DNA damage induced by etoposide was increased in BRCA1-null compared with BRCA1+ cells (Supplementary Fig. 1).

**Fig. 1:**
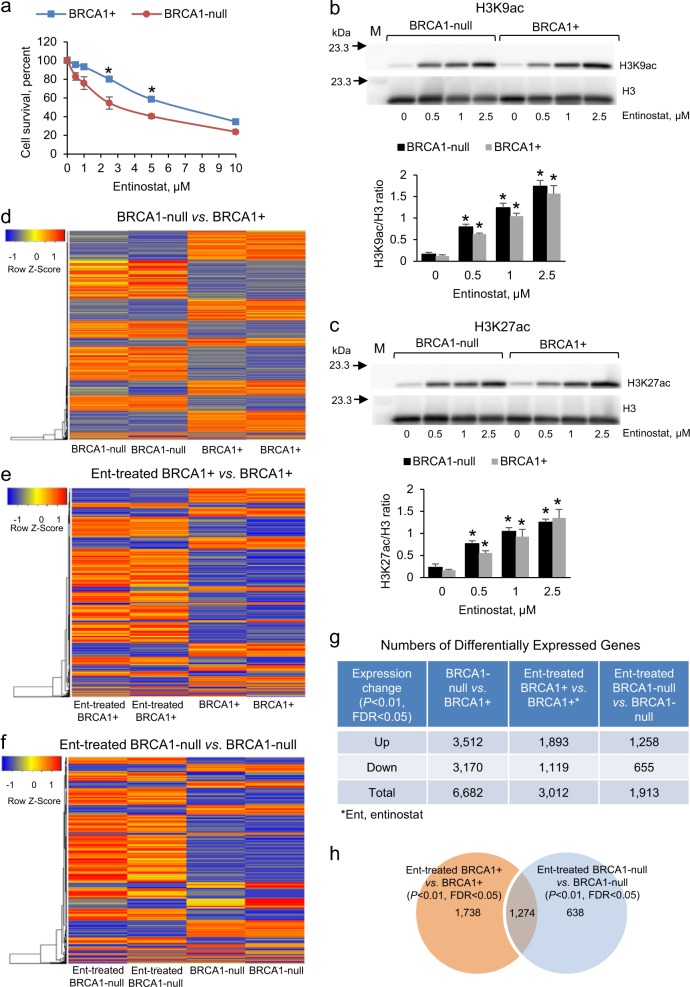
Gene expression changes associated with BRCA1 mutation in ovarian cancer (OC) cells. **a** Survival (means ± s.e.m., *n* = 4) measured by the CCK8 assay of UWB1.289 OC cells (BRCA1-null) and UWB1.289 cells transfected with BRCA1 (BRCA1+) and treated with entinostat for 72 h. *P < 0.05 (*t*-test). **b**, **c** Western blot and densitometric analysis (means ± s.e.m., *n* = 3) of entinostat effects (72 h treatment) on H3K9ac **b** and H3K27ac **c** levels in BRCA1-null and BRCA1+ UWB1.289 OC cells. *P < 0.05 (*t*-test) relative to 0 entinostat. M, protein markers. **d**–**f** Unsupervised hierarchical clustering of differentially expressed genes measured by RNAseq between BRCA1-null and BRCA1+ **d**, entinostat-treated BRCA1+ compared with BRCA1+ **e**, and entinostat-treated BRCA1-null compared with BRCA1-null **f** UWB1.289 OC cells. Colored horizontal lines represent genes and columns represent specimens analyzed. Cells were treated with 0.5 μM entinostat for 24 h (*n* = 2). **g** Numbers of differentially expressed genes in BRCA1-null relative to BRCA1+UWB1.289 OC cells treated with 0.5 μM entinostat for 24 h (*n* = 2). **h** A Venn diagram shows numbers of common and unique differentially expressed genes determined by RNAseq in BRCA1+ and BRCA1-null UWB1.289 OC cells treated with 0.5 μM entinostat (Ent) for 24 h (*n* = 2).

We observed no differences in cell survival after treatment with either a DNA methyltransferase inhibitor (guadecitabine; Supplementary Fig. 2a) or an inhibitor of the polycomb repressive complex 2 enzymatic component EZH2 (GSK126; Supplementary Fig. 2b) between OC cells carrying a deleterious BRCA1 mutation and those with functional BRCA1. However, BRCA1-null cells were more sensitive than BRCA1+ cells to the HDAC inhibitor entinostat (Fig. 1a; IC_50_ of 3.0 μM vs. 6 μM), suggesting potential baseline differences in histone protein acetylation between the two cell lines. Total HDAC activity and *HDAC1* expression levels were also slightly decreased in BRCA1-null vs. BRCA1+ cells (Supplementary Fig. 3a, b), while H3K9ac and H3K27ac basal levels were modestly increased in BRCA1-null vs. BRCA1+ cells (Supplementary Fig. 3c, d). Consistent with its known HDAC inhibitory activity, entinostat induced H3K9 and H3K27 acetylation relative to H3 levels in both cell lines (Fig. 1b, c).

### Transcriptomic changes associated with mutated BRCA1 and response to an HDAC inhibitor

We next examined changes in gene expression after HDACi treatment. Entinostat, an HDAC1 and HDAC3 inhibitor, was used at 0.5 μM (non-toxic concentration). Unsupervised hierarchical clustering (Fig. 1d–f) and principal component analysis (PCA, Supplementary Fig. 4) demonstrated differences in gene expression associated with BRCA1 deficiency (Fig. 1d) and in response to entinostat in BRCA1+ (Fig. 1e) and BRCA1-null (Fig. 1f) cells. Absence of a functional BRCA1 was associated with changes in expression (*P* < 0.01, *t*-test, FDR < 0.05) of more than 6000 genes (BRCA1-null vs. BRCA1+, Fig. 1g). BRCA1 deficiency also altered the response to entinostat; 1738 and 638 genes whose expression was changed by entinostat were unique to BRCA1+ or BRCA1-null cells, respectively, while expression of 1274 genes changed in response to the HDACi in both cell lines (Fig. 1h, Supplementary Tables 3–5).

### Distinct gene expression profiles associated with BRCA1 expression levels and mutational status in HGSOC

**Fig. 2:**
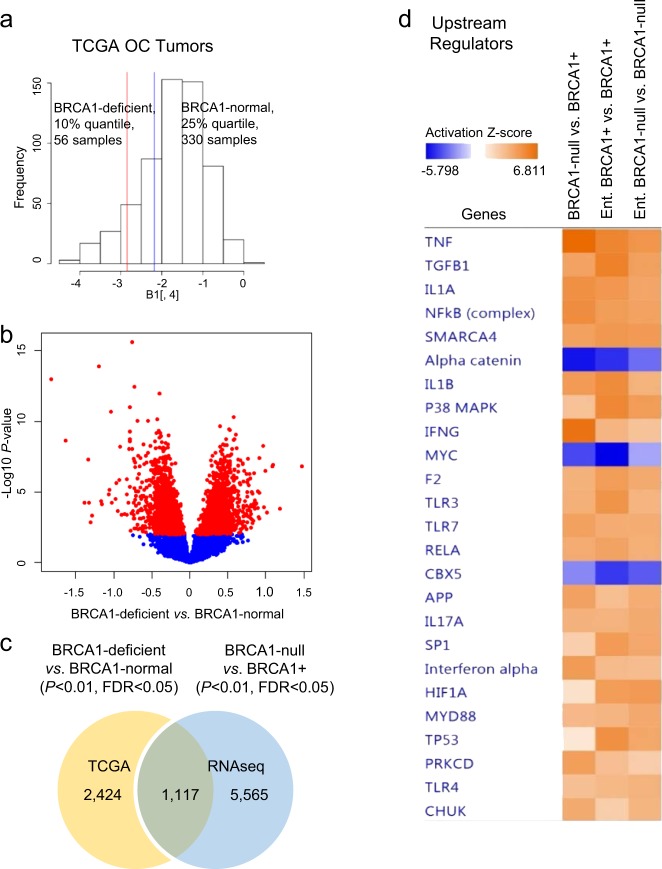
Gene expression profiles associated with *BRCA1* expression levels in ovarian cancer (OC) tumors. **a** Distribution of *BRCA1* mRNA expression levels in high-grade serous ovarian cancer (HGSOC) specimens from the TCGA dataset. The upper 25% quantile (blue line to the right) were considered BRCA1-normal while the low 10% quantile (red line to the left) were considered BRCA1-deficient. **b** Volcano plot of differentially expressed genes between BRCA1-deficient and BRCA1-normal HGSOC tumors in the TCGA database. **c** Venn diagram shows the numbers of specific or shared genes differentially expressed between BRCA1-deficient and BRCA1-normal OC tumors (TCGA) and between BRCA1-null and BRCA1+ UWB1.289 OC cells. **d** IPA comparative analysis shows activation *Z*-scores of differentially expressed upstream regulators in BRCA1-null relative to BRCA1+ UWB1.289 OC cells, and in the same cell lines in response to entinostat treatment (0.5 μM for 24 h, *n* = 2).

To determine whether the genomic changes observed between BRCA1 mutant and WT cells correlate with molecular profiles of human tumors, we analyzed gene expression measured by Agilent G4502A microarrays in HGSOC tumors from the TCGA^1^ program. These tumors were grouped based on BRCA1 expression levels into BRCA1-deficient (56 tumors, BRCA1 expression <10th quantile) or mutated (19 tumors) and BRCA1-normal (330 tumors, BRCA1 expression >25th quantile), as described in Fig. 2a. Expression levels of 3541 genes differed significantly (*P* < 0.01, *t*-test, FDR < 0.05) between BRCA1-deficient or mutated and BRCA1-normal tumors (Fig. 2b, volcano plot), indicating that the expression and function of BRCA1, directly or indirectly, induce changes in the transcriptome of HGSOC. Additionally, 1117 genes were differentially expressed both in BRCA1-null vs. BRCA1+ OC cells and in the BRCA1-deficient vs. BRCA1-normal ovarian tumors (Fig. 2c), supporting similarity between the selected cellular model to study BRCA1 mutation-associated transcriptomic changes and human HGSOC tumors.

### BRCA1 deficiency was associated with changes in gene networks regulated by entinostat in OC cells and tumors

To gain further insight into the functional implications of transcriptomic differences in BRCA1-mutated cells and tumors, we performed Ingenuity Pathway Analysis (IPA, Supplementary Tables 6 and 7) and compared the “Top Molecular and Cellular Functions” (Table 1) and the “Top Upstream Regulators” (Table 2) enriched in BRCA1-null/deficient vs. BRCA1+ cells and tumors. Two functions (cell-to-cell signaling and interaction, and cellular growth and proliferation, and two upstream regulators (TGFB1 and IFNG) were shared between differentially expressed genes in the isogenic cell lines and differentially expressed genes in BRCA1-deficient vs. normal ovarian tumors. Comparison of upstream regulators activated or inhibited in the analysis comparing BRCA1-null vs. BRCA1+ cells, entinostat-treated BRCA1+, and entinostat-treated BRCA-null cells showed changes induced by entinostat in the activation *Z*-score of several upstream regulators (e.g. *IFNG, TNF*, *NF-kB*, *IL1A*, *TGFB1*, and others; Fig. 2d). *IFNG* was detected as activated (*Z*-score 6.7) in BRCA1-null relative to BRCA1+ cells, and also in entinostat-treated BRCA1+ vs. BRCA1+ (*Z*-score 4.0), but not in entinostat-treated BRCA-null vs. BRCA1-null cells (*Z*-score <2). This suggested that activation of the *IFNG* pathway could be induced by HDAC inhibition in BRCA1+ cells to levels similar to those observed in BRCA1-null cells, and that this effect requires the presence of a functional BRCA1. Other upstream regulators such as *TNF* and *TGFB1* were activated by entinostat regardless of BRCA1 status. These integrated analyses indicated that the *IFNG* pathway in particular was influenced by BRCA1 loss of function and was altered in response to HDACi in OC cells and tumors expressing functional BRCA1.

**Table 1:** Top molecular and cellular functions identified by IPA among genes differing in expression (RNAseq, left) between BRCA1-null and BRCA1+ ovarian cancer (OC) cells, and among differentially expressed genes between BRCA1-deficient and BRCA1-normal OC tumors from the TCGA database (right).

BRCA1-null vs. BRCA1+ OC cells			BRCA1-deficient vs. BRCA1-normal OC tumors, TCGA		
Molecular and cellular functions	*P* value	Number of molecules	Molecular and cellular functions	*P* value	Number of molecules
Cellular movement	5.3E-10–1.6E-53	702	Cellular growth and proliferation	1.6E-03–2.1E-11	975
Cellular development	5.1E-10–4.8E-37	995	Cell death and survival	2.6E-03–1.1E-09	966
Cellular growth and proliferation	5.1E-10–8.4E-31	1057	Cellular function and maintenance	2.5E-03–5.1E-07	770
Cell morphology	5.1E-10–1.7E-30	721	Cell-to-cell signaling and interaction	2.6E-03–9.7E-07	324
Cell-to-cell signaling and interaction	3.7E-10–4.8E-30	588	Molecular transport	2.4E-03–1.1E-06	574

**Table 2:** Top upstream regulators determined by IPA among genes differing in expression between BRCA1-null and BRCA1+ OC cells (left), and among differentially expressed genes between BRCA1-deficient and BRCA1-normal OC tumors from the TCGA database (right).

BRCA1-null vs. BRCA1+ ovarian cancer cells			BRCA1-deficient vs. BRCA1-normal ovarian cancer tumors, TCGA	
Top upstream regulator	*P* value of overlap	Predicted activation	Top upstream regulator	*P* value of overlap
TNF	2.1E-61	Activated	HNF4A	7.4E-19
TGFB1	3.3E-51	Activated	TP53	1.8E-10
IFNG	1.4E-36	Activated	TGFB1	1.4E-09
β-Estradiol	1.8E-35		FAS	7.8E-08
SMARCA4	2.2E-33	Activated	ESR1	5.9E-07
IL1B	2.6E-33	Activated	Interferon beta-1a	1.7E-06
Progesterone	2.2E-27		IFNG	4.6E-06
Estrogen receptor	2.1E-24	Inhibited	OSM	4.7E-06
SP1	1.6E-23	Activated	FOXO1	6.9E-06
VEGF	9.0E-23	Activated	IL27	1.6E-05

### Activation of IFN-γ pathway in BRCA1-null vs. BRCA1+ cells

**Fig. 3:**
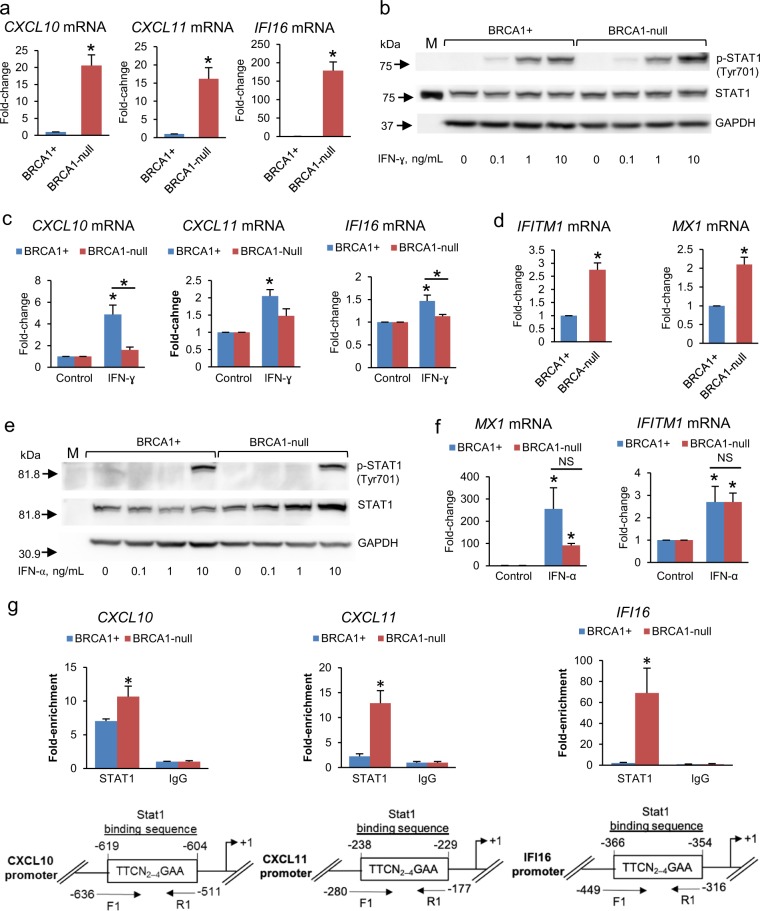
Expression of IFN-regulated genes in BRCA1-null and BRCA1+ cancer cells. **a** *mRNA* expression levels (means ± s.e.m., *n* = 3) measured by qRT-PCR of the IFN-γ-regulated genes *CXCL10*, *CXCL11*, and *IFI16* in BRCA1-null UWB1.289 OC cells with BRCA1 restored (BRCA1+) or transfected with empty vector (BRCA1-null). *P < 0.01 (*t*-test). **b** Western blot measures STAT1 and p-STAT1 in BRCA1+ and BRCA1-null UWB1.289 OC cells treated with IFN-γ for 15 min. M, protein markers. **c** Expression levels (means ± s.e.m., *n* = 3) of the IFN-γ-regulated genes *CXCL10, CXCL11*, and *IFI16* in BRCA1+ and BRCA1-null OC cells treated with 10 ng/mL IFN-γ for 24 h. *P < 0.05 (*t*-test) relative to control (0 dose) or between cell lines as indicated. **d** *mRNA* expression levels (means ± s.e.m., *n* = 3) of the IFN-α-regulated genes *IFITM1* and *MX1* in BRCA1+ and BRCA1-null UWB1.289 OC cells. *P < 0.05 (*t*-test). **e** Measurements by western blot of STAT1 and p-STAT1 in BRCA1+ and BRCA1-null UWB1.289 OC cells treated with IFN-α for 15 min at the indicated doses. **f** Expression levels (means ± s.e.m., *n* = 3) of the IFN-α-regulated genes *MX1* and *IFITM1* in BRCA1+ and BRCA1-null UWB1.289 OC cells treated with 10 ng/mL IFN-α for 24 h. *P < 0.05 (*t*-test) relative to control (0 dose) or between cell lines as indicated. **g** Binding levels (means ± s.e.m., *n* = 3) of STAT1 to the promoters of IFN-γ-regulated genes *CXCL10*, *CXCL11*, and *IFI16* measured by ChIP in BRCA1+ and BRCA1-null UWB1.289 OC cells. *P < 0.05 (*t*-test); F1 forward primer; R1 reverse primer.

We next verified whether the *IFNG* pathway was indeed activated in BRCA1-null vs. BRCA1+ cells. *IFI44, IRF9, IL1A, OAS2*, and *OAS3* were identified among 17 IFN-γ-regulated genes classified by IPA as been activated (Supplementary Table 8). To validate activation of the IFN-γ pathway in the presence of a BRCA1 mutation, we measured basal and IFN-γ-induced expression of several target genes in BRCA1-null cells (stably transfected with the vector pcDNA3.1) and in BRCA1+ UWB1.289 OC cells. Among the key target genes, the inflammatory chemokines encoded by *CXCL10* (C-X-C motif chemokine 10) and *CXCL11* (C-X-C motif chemokine 11) promote T cell chemotaxis and play a role in anti-tumor immunity.^28^ *IFI16* (interferon gamma inducible protein 16) is an innate sensor for intracellular DNA that triggers a pro-inflammatory and growth inhibitory response through activation of IFN-β and NF-κB pathways.^29–31^ Basal levels of *CXCL10* (~20-fold), *CXCL11* (~15-fold), and *IFI16* (~160-fold) were significantly upregulated in BRCA1-null (+vector) vs. BRCA1+ OC cells (Fig. 3a). IFN-γ induced a more robust phosphorylation of STAT1 in BRCA1-null (+vector) cells compared to BRCA1+ cells (Fig. 3b, Supplementary Fig. 5a); however, induced expression of *CXCL10* and *IFI16* (Fig. 3c) was less in BRCA1-null (+vector) vs. BRCA1+ cells, probably because baseline levels of these transcripts were already significantly increased in BRCA1-null cells. In contrast, differences in responses to IFN-α were less pronounced. Baseline expression levels of *IFITM1* and *MX1*, known IFN-α targets,^21^ were also increased in BRCA1-null (+vector) compared to BRCA1+ cells, but to a lesser extent than IFN-γ-regulated genes (Fig. 3d), while the IFN pathway inhibitor *suppressor of cytokine signaling* 2 (*SOCS2*) was downregulated in BRCA1-null cells (not shown), perhaps contributing to activation of the IFN pathway in this context. Response to IFN-α stimulation was similar in the two cell lines as measured by STAT1 phosphorylation at Tyr701 (Fig. 3e, Supplementary Fig. 5b) and target gene induction (Fig. 3f), although no differences in basal STAT1 phosphorylation were detected between the cell lines.

To understand the mechanism causing activated basal IFN-γ signaling in BRCA1-null cells, we assessed the recruitment of the transcription factor STAT1 to the promoters of target genes *CXCL10*, *CXCL11*, and *IFI16* by using chromatin immunoprecipitation (ChIP). Increased STAT1 localization in regions containing the predicted STAT1-binding sites (GAS elements) within the promoters of *CXCL10*, *CXCL11*, and *IFIT16* was observed in BRCA1-null vs. BRCA1+ cells (Fig. 3g), in the absence of ligand. These data support the concept that the pathway is activated under basal conditions in cells carrying a BRCA1 mutation.

**Fig. 4:**
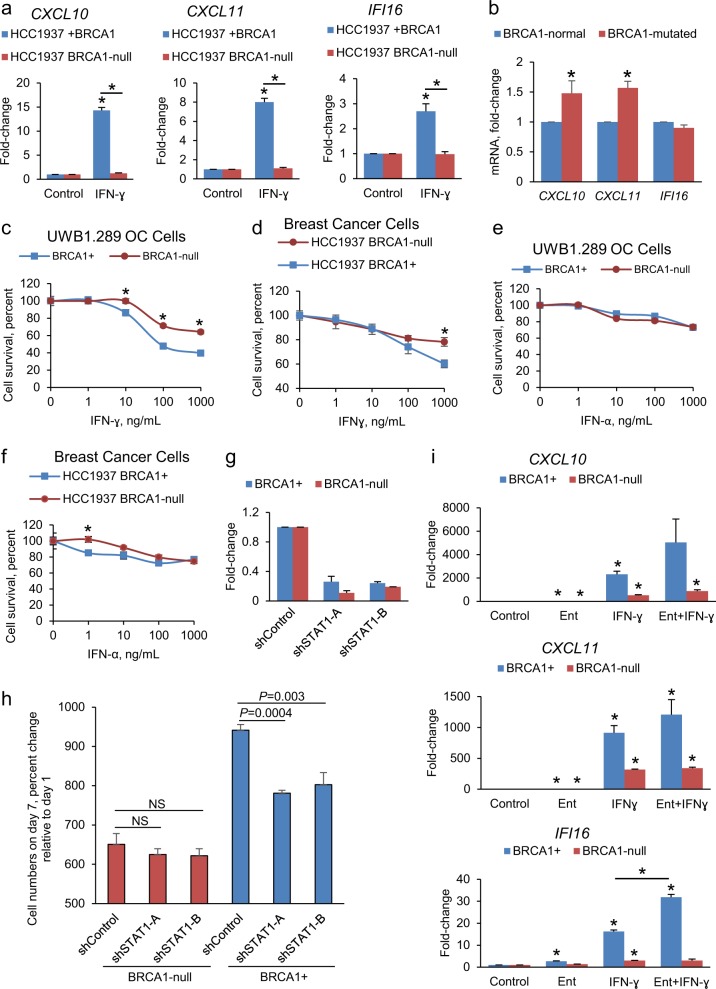
Cellular responses to IFN-γ depend upon BRCA1 mutational status. **a** Expression levels (means ± s.e.m., *n* = 3) of the *CXCL10, CXCL11*, and *IFI16* genes measured by qRT-PCR in HCC1937 (BRCA1-null) and HCC1937+ BRCA1 breast cancer (BC) cells treated with IFN-γ for 24 h. *P < 0.05 (*t*-test) relative to control (0 h) or between cell lines as indicated. **b** *CXCL10*, *CXCL11*, and *IFI16* mRNA expression levels in BRCA1-normal compared with BRCA1-mutated immortalized mammary epithelial cells. *P < 0.05 (mean ± s.e.m. of three independent measurements in two BRCA1-normal and one BRCA1-mutated cell cultures, *t*-test). **c**–**f** Cell survival (mean ± SE, *n* = 4) measured by the CCK8 assay of BRCA1-null and BRCA1+ ovarian cancer (OC) or BC cells treated with IFN-γ (**c**, **d**) or with IFN-α (**e**, **f**) for 96 h. *P < 0.05 (*t*-test) between groups at the indicated doses. **g** *STAT1* mRNA levels (means ± s.e.m., *n* = 2) measured by qRT-PCR in BRCA1+ and BRCA1-null UWB1.289 OC cells transduced with a non-targeting shRNA (shControl) or two different shRNAs directed at *STAT1*. **h** CCK8 assay measured proliferation of BRCA1+ and BRCA1-null UWB1.289 OC cells transduced with shControl or shRNAs targeting *STAT1*. NS not different (*P* > 0.05, *t*-test). Values are percentage of cells (means ± s.e.m., *n* = 4) relative to day 1. Shown are *t*-test *P* values. **i** Effects of entinostat (Ent, 0.5 μM), IFN-γ (10 ng/mL), or combination treatment for 24 h on mRNA expression levels (means ± s.e.m., *n* = 3) of the *CXCL10, CXCL11*, and *IFI16* genes in BRCA1+ and BRCA1-null UWB1.289 OC cells. *P < 0.05 (*t*-test) relative to Control or between BRCA1+ treated with IFN-γ vs. Ent+ IFN-γ for *IFI16*.

To exclude the possibility that these observations were cell model dependent, we tested the response to IFN in isogenic BRCA1 mutated breast cancer cells (HCC1937) and cells stably transfected with BRCA1 (HCC1937+ BRCA1). BRCA1 protein was absent in HCC1937 compared to HCC1937+ BRCA1 cells, consistent with their known phenotype^32^ (Supplementary Fig. 5c). IFN-γ-induced expression of *CXCL10, CXCL11, IFI16* (Fig. 4a), *MX1*, and *IFITM1* (Supplementary Fig. 5d) was abrogated in BRCA1-mutated HCC1937 cells compared with HCC1937+ BRCA1 cells to an even greater extent than observed levels in OC cells. To verify the increased activation levels of the IFN-γ pathway associated to BRCA1 mutations, we measured expression of IFN-γ-regulated genes in other cellular models and tumors. Expression of *CXCL10* and *CXCL11* was increased in BRCA1 mutated vs. BRCA1-normal mammary immortalized cells lines, as measured by qRT-PCR (Fig. 4b), and in BRCA1-deficient relative to BRCA1-normal OC tumors from TCGA (Supplementary Fig. 6a). Together, these data support that BRCA1-mutated cells display elevated IFN-γ activation and respond less efficiently to IFN-γ than cells harboring a functional BRCA1.

### BRCA1 mutational status alters cellular responses to IFN-γ

We next determined the cellular responses to IFNs in isogenic BRCA1+/− cells. IFN-γ decreased (*P* < 0.05, *t*-test) the survival of both cell types, with increased cytotoxic effects in BRCA1+ vs. BRCA1-null cells in both OC and BC cell models (Fig. 4c, d). In contrast, IFN-α had modest effects on the survival of BRCA1-null or BRCA1+ OC cells, even at concentrations of 1 μg/mL (Fig. 4e, f). Furthermore, shRNA mediated stable knock down of STAT1 (Fig. 4g), the main transcription factor mediating IFN-γ signaling, significantly suppressed the proliferation of BRCA1+ cells, but did not impact BRCA1-null OC cells (Fig. 4h), supporting that cells carrying a BRCA1 mutation were less susceptible to IFN-γ-mediated cytotoxicity. Expression levels of *IFI16* were downregulated by STAT1 knock down in BRCA1+, but not in BRCA1-null cells, suggesting less responsiveness to this pathway manipulation in cells harboring deleterious BRCA1; however, the other two IFNγ targets (*CXCL10* and *CX*CL11) were not consistently altered by STAT1 knock down (Supplementary Fig. 6b).

Having observed activation of the *IFNG* pathway in response to entinostat in our RNA-sequencing analyses in cells with functional BRCA1 (Fig. 2d) and based on previous observations suggesting cooperation between STAT1 and HDAC1 in transcription regulation,^33^ we investigated responses to IFNs in the presence of an HDACi in BRCA1-null vs. + cells. Interestingly, the response to IFN-γ (*CXCL10*, *CXCL11*, and *IFI16 mRNA* expression) was augmented by entinostat in BRCA1+, but BRCA1-null cells remained resistant to IFN-γ, even after treatment with HDACi (Fig. 4i), supporting that regulation of IFN-γ responses is influenced by BRCA1 and histone deacetylation. Addition of either IFN-α or IFN-γ (10 ng/mL) to entinostat did not increase cytotoxicity in BRCA1+ cells (Supplementary Fig. 7a), but both IFNs potentiated the cytotoxic effects induced by entinostat in BRCA1-null OC cells (Supplementary Fig. 7b). The effects of entinostat on key genes involved in IFNγ signaling in BRCA1-null and BRCA1+ cells, as measured by RNA-sequencing, are summarized in Supplementary Table 9. Collectively these data support that HDAC inhibition alters the cellular responses to IFNs and that these effects depend on functional BRCA1.

### Genome-wide histone H3 acetylation differs in BRCA1-null and BRCA1+ cells

**Fig. 5:**
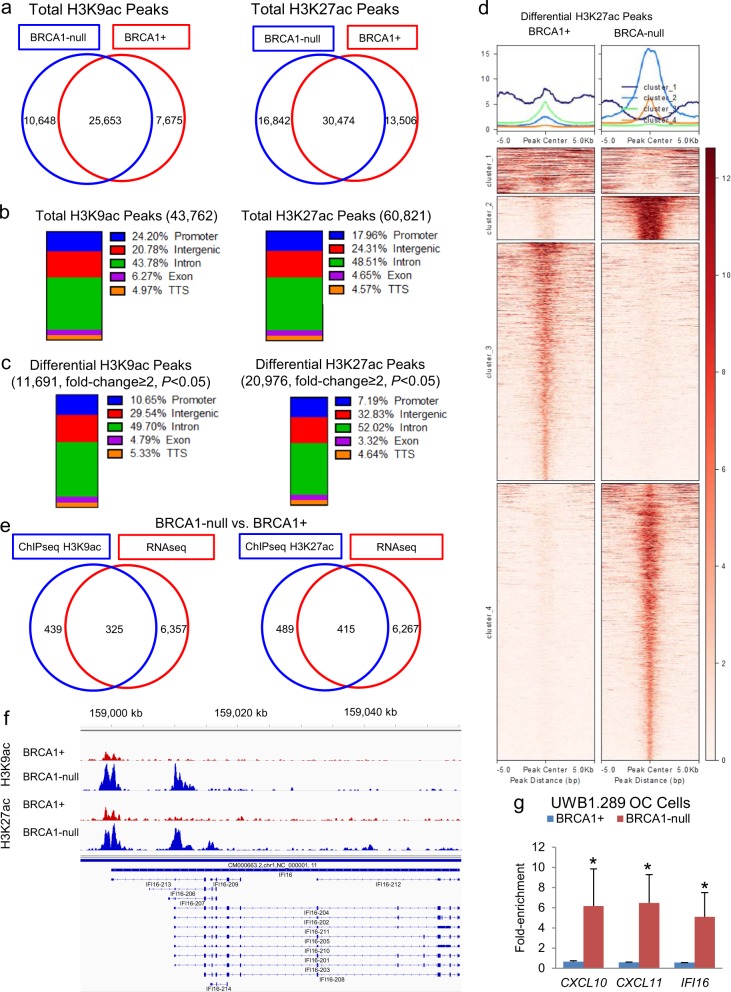
Differential histone H3K9 and H3K27 acetylation in ovarian cancer (OC) cells carrying a BRCA1 loss-of-function mutation (BRCA1-null). **a** Venn diagrams illustrate numbers of unique and common H3K9ac or H3K27ac peaks in BRCA1-null and BRCA1+ UWB1.289 OC cells measured by ChIP-seq. **b** Percent distribution of all H3K9ac or H3K27ac peaks among genomic regions in BRCA1+ and BRCA1-null UWB1.289 OC cells. **c** Percent distribution among genomic regions of differential H3K9ac or H3K27ac peaks in BRCA1-null vs. BRCA1+ UWB1.289 OC cells. **d** Differential H3K27ac peaks (−5000 bp to +5000 bp from the peak center) grouped into four clusters and represented as integrated plots (top) or a heatmap (bottom) in BRCA1-null vs. BRCA1+ UWB1.289 OC cells. Rows on the heatmap represent H3K27ac peaks. **e** Venn diagrams shows numbers of common genes having differential promoter H3K9ac or H3K27ac marks (ChIP-seq, blue) and differential expression (RNAseq, red) between BRCA1-null and BRCA1+ UWB1.289 OC cells. **f** Profiles of H3K9ac and H3K27ac peaks in the IFN-γ-regulated gene *IFI16* in BRCA1-null vs. BRCA1+ UWB1.289 OC cells. **g** H3K9ac enrichment (means ± s.e.m., *n* = 2) measured by ChIP-PCR in the promoters of the IFN-γ-regulated genes *CXCL10*, *CXCL11*, and *IFI16* in BRCA1-null and BRCA1+ UWB1.289 OC cell line *P < 0.05 (*t*-test).

Based on the observed differences in response to HDACi, we examined differential H3K9 and H3K27 acetylated chromatin marks (basal state) between BRCA1-null and BRCA1+ cells. ChIP-seq analysis identified more than 100,000 peaks corresponding to H3K9ac or H3K27ac marks in the genome, with nearly half of them located in introns (Fig. 5a, b). More H3K9ac and H3K27ac marks (2973 and 3336, respectively) were identified in BRCA1-null than in BRCA1+ cells (Fig. 5a) corroborating greater H3K9 and H3K27 acetylation levels in the absence of BRCA1 measured by western blotting (Supplementary Fig. 3c, d). A total of 11,691 H3K9ac and 20,976 H3K27ac marks differed between BRCA1-null vs. BRCA1+ cells, approximately half of them located in introns, 30% in intergenic, and the remaining in promoters, exons, and TSS genomic regions (Fig. 5c). Clustering of differential H3K27ac peaks, which are usually associated with enhancers and promoters, showed increased acetylation within two clusters in BRCA1-null cells but not in the other clusters (Fig. 5d). We identified 325 gene promoters differentially marked with H3K9ac and 415 with H3K27ac, which were associated with differential gene expression (RNA-seq) in BRCA1-null vs. BRCA1+ cells (Fig. 5e); and among them, we detected the IFN-γ-regulated gene *IFI16* (Fig. 5f). Increased deposition of H3K9ac in the promoter region of *IFI16* in RBCA1-null vs. BRCA1+ UWB1.289 OC cells was also verified by real-time PCR on chromatin immunoprecipitated with H3K9ac antibody (Fig. 5g, Supplementary Fig. 8). These data support the notion that IFN-γ signaling in the BRCA1-null OC cells is associated with distinct deposition of H3K9 and H3K27 acetylated marks.

### IFN-γ signature in HGSOC

**Fig. 6:**
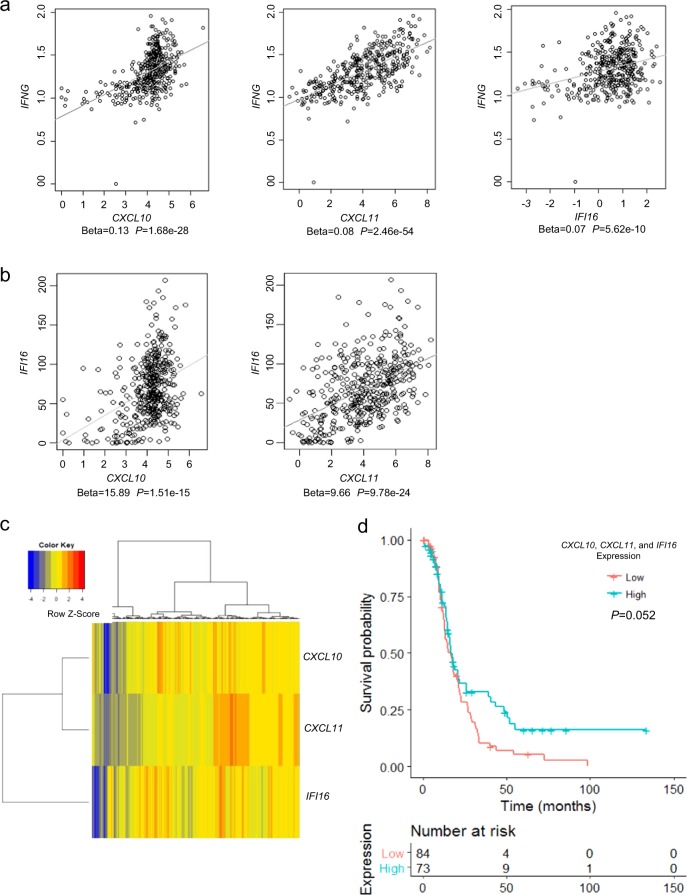
IFN-γ signaling in high-grade serous ovarian cancer (HGSOC) tumors. **a**, **b** Associations determined by regression analysis between mRNA levels of *CXCL10*, *CXCL11*, or *IFI16* with IFN-γ **a**, and between *IFI16* with *CXCL10* or *CXCL11* **b** in HGSOC samples from the TCGA database. **c** Hierarchical clustering of HGSOC tumor specimens profiled in the TCGA based on *CXCL10*, *CXCL11*, and *IFI16 mRNA* expression levels identified two groups of patients (samples). Columns correspond to individual tumors. **d** Disease-free survival analysis for patients profiled in the TCGA and grouped as having “low” or “high” (relative to their median values) combined expression levels for the IFN-γ-regulated genes *CXCL10*, *CXCL11*, and *IFI16*. The *P* value is for Cox proportional hazard regression analysis.

To assess the significance of the IFN-γ pathway in human tumors, we used regression analysis and measured the association between *mRNA* levels of IFN-γ and *CXCL10, CXCL11*, or *IFI16* in HGSOC tumors from the TCGA database. Statistically significant associations between IFN-γ and each of the three genes were observed, supporting that *CXCL10, CXCL11*, and *IFI16* are inducible by IFN-γ in HGSOC (Fig. 6a). Additionally, the *mRNA* expression levels of *CXCL10*, or *CXCL11* with *IFI16* were highly positively correlated (*P* < 1 × 10^−15^, Fig. 6b), and separated the tumors in two distinct subgroups, as shown by hierarchical clustering (Fig. 6c). To determine whether the IFN-γ signature carried prognostic value, OC samples were classified as “high expressing” subgroup, if the expression of the three genes was higher than their corresponding median values or as “low expressing” subgroup, if the expression level for all three genes was lower than the median value. Seventy-three and 84 ovarian tumors were thus classified as “high” and “low” subgroups and a trend towards prolonged overall survival recorded for the IFN-γ “high” vs. “low” subgroups (Fig. 6d, *P* = 0.052, Cox proportional hazard regression). Taken together, the data support the clinical significance of IFN-γ and the potential loss of sensitivity to IFN-γ in BRCA1-null tumors due to a baseline augmented status.

Lastly, given the significance of the IFN-γ pathway to anti-tumor immune responses and to determine whether BRCA1-deficient and normal tumors differed in type of immune cell infiltrates, we used TIMER to predict the relative numbers of immune cells in tumor specimens based on gene expression data.^34^ Predicted numbers of six immune cell subtypes (B cells, CD4, CD8, macrophages, neutrophils, and dendritic cells) are shown in Supplementary Fig. 9. Significant differences in neutrophils (*P* *=* 0.001, *t*-test) and dendritic cells (*P* *=* 0.01, *t*-test) infiltration were estimated based on the transcriptomic signature for BRCA1-deficient vs. normal ovarian tumors, but no differences were detectable for the other immune cell types.

## Discussion

Consistent with previous reports,^14,15^ our findings support that BRCA1 mutations are associated with significant transcriptomic changes in OC cells and tumors. BRCA1 has been implicated in transcriptional regulation through several mechanisms including direct interactions with the p300/CBP activator complex^35^ and maintenance of the heterochromatin structure through ubiquitylation of histone H2A.^36^ Here we show that some of these changes are associated with distinct patterns of chromatin-associated H3K9 and H3K27 acetylated marks, leading to differences in cellular responses to HDACi and to an IFN-γ gene signature detectable in BRCA1-mutated cancer cells. This signature was also validated in the gene expression profiles of ovarian carcinomas profiled by the TCGA.^1^ Our results have several implications.

First, we observed differences in cell proliferation in response to treatment with HDACi in cells carrying a BRCA1 loss of function mutation vs. cells expressing functional BRCA1. Interestingly, HDACi have been recently shown to downregulate expression of genes involved in DNA damage response (*RAD51* and *BRCA1*)^17^ and to sensitize homologous recombination proficient cells to DNA damage-inducing agents. It is therefore possible that the increased cytotoxicity of entinostat observed in BRCA1 mutated cancer cells was caused by the impairment in the DNA damage response in addition to the homologous recombination deficiency caused by BRCA1 loss of function. However, the mechanism by which genes involved in DNA repair are downregulated by HDACi remains not clear.

Here we show distinct transcriptomic profiles corresponding to distinct mapping of H3K9ac and H3K27ac depending on the presence of a functional BRCA1 and leading to activation of specific gene networks in BRCA1-null cells and tumors. A potential explanation for these findings is the previously recognized interaction between BRCA1 and elements of the histone deacetylation complex, including HDAC1 and 2 and the *Retinoblastoma* (*Rb*)-associated proteins RbAp46 and RbAp48.^19^ This interaction was mediated by the BRCT domain, which is truncated in the majority of BRCA1-mutated ovarian and breast tumors, including in the cellular models used in the current study, suggesting that the BRCT domain may be indirectly implicated in protein–protein interactions leading to chromatin remodeling and transcription regulation. In support of this concept, co-recruitment of BRCA1 and HDAC to the promoter of target genes has been documented for progesterone receptor (*PR*) target genes, BRCA1 acting as a repressor by displacing the coactivator *AIB1* and recruiting the co-repressor *HDAC1* at the PR responsive elements.^37^ Interestingly, we observed decreased HDAC1 enzymatic activity in nuclear extracts from BRCA1-null vs. BRCA1+ cells in association with differences in acetylated chromatin marks between the isogenic OC cell lines, corroborating previous evidence linking BRCA1 and histone deacetylases.

Secondly, we detected an augmented baseline IFN-γ signature in BRCA1-mutated cancer and primary epithelial cells and tumors coupled with decreased cytotoxicity induced by IFN-γ or by STAT1 knockdown. Of the IFN-γ-related genes, *IFI16* was upregulated >150-fold at baseline in BRCA1-mutated cancer cells. We observed increased H3K9Ac and H3K27Ac peaks at the promoter and enhancer regions of this gene in BRCA1-mutated OC cells, supporting its transcriptional activation. IFI16 is a member of the PYHIN family which comprises a pyrin domain and two DNA-binding HIN domains. IFI16 was shown to act as an intracellular sensor for viral DNA, to directly bind and activate the STING pathway, leading to IFN response.^31^ It is possible that in conditions of genomic instability related to the presence of a non-functional BRCA1, this pathway is activated possibly by the presence of intracellular DNA, initiating an IFN response.

Interestingly, differential apoptotic response to IFN-γ dependent on the presence of functional BRCA1 was previously observed in the HCC1937 isogenic breast cancer model,^38^ although the mechanism responsible for this phenomenon was not elucidated. Similarly, it had been reported that IRF7 expression in response to IFN-γ was augmented in the presence of functional BRCA1, in a manner dependent of STAT1 and STAT2,^39^ consistent with our observations documenting a blunted response to IFN-γ in BRCA1-deficient cells. Finally, a direct interaction between the BRCT domain of BRCA1 and STAT1 has been implicated in induction of p21 and apoptosis in cancer cells in response to IFNs.^40^ We did not identify differences in IFN-γ receptor expression or ligand secretion between the isogenic cells lines and have attributed the augmented IFN response to an altered transcriptomic program linked to redistributed H3 acetylated marks in the presence of BRCA1 mutations. A recent report showed that treatment with HDAC and DNA methylation inhibitors triggered an IFN response in a lung cancer model,^41^ supporting the hypothesis that regulation of this pathway is highly dependent on histone deacetylation. This concept is also supported by our observations showing that addition of an HDACi significantly increased cellular responses to IFNs in BRCA1+ cells.

Thirdly, our observations that BRCA1-mutated cells are resistant to IFN-γ-mediated cytotoxicity and to STAT1 downregulation could have implications for understanding responses to anti-tumor immunity in BRCA1-mutated cancers. These results could suggest that this mechanism may play a role in tumor initiation and progression related to BRCA1 loss of function, as cancer cells lacking this TSG could use this mechanism to escape the host’s anti-tumor immune surveillance mechanisms. Further, these observations could fuel speculations that BRCA1-null tumors could be resistant to immune interventions. Interestingly, an *mRNA* based IFN-γ signature was found to be predictive of response to immune checkpoint inhibitors in a panel of cancer patients treated with pembrolizumab^42^ and mutations in the JAK/STAT pathway were linked to resistance to immunotherapy.^43^ While OC is not highly immunogenic, increased levels of immune effector cells within the tumor have correlated with better anti-tumor responses and improved survival.^44,45^ It had been speculated that BRCA-deficient tumors may be associated with a more robust anti-tumor immunity, as suggested by an increased T cell infiltration compared to tumors harboring intact BRCA1 (refs ^46,47^) and perhaps by an increased somatic mutation load in these genomically unstable tumors. Our exploratory transcriptomic analyses using TIMER predicted that dendritic cells and neutrophils as being differentially present in BRCA1-deficient vs. BRCA normal ovarian tumors, also supporting the concept that cellular immune responses are augmented in this setting. However, these findings have not yet panned out in clinical trials; in a recent evaluation of avelumab in patients with ovarian cancer, clinical responses to this anti PD-L1 antibody were uncommon in both patients with BRCA1/2 mutated or in those with BRCA1/2 wild-type tumors.^48^ Additionally, a recent preclinical study using an immune-competent mouse model harboring BRCA1-mutated tumors reported lack of response to immune checkpoint inhibition.^49^ Thus, our results showing that BRCA1-null cells are less susceptible to the cytotoxic effects of IFN-γ and STAT1 knock down are hypothesis generating and could argue that such cells may resist elimination by immune cell-induced IFNγ response due to an enhanced baseline IFNγ signature. However, in the tumor microenvironment other mechanisms may be operative and able to overcome this pathway. Future analyses of tissue specimens from clinical trials will be necessary to fully understand sensitivity or resistance to immune interventions in this context.

Lastly, we show that the combination of high *mRNA* expression levels for three IFN-γ target genes (*CXCL10, CXCL11 and IFI16*) correlated with overall survival in ovarian tumors from the TCGA database, pointing to the potential clinical significance of this pathway in HGSOC. In all, these conclusions led us to propose that tumor-related BRCA1 mutations are associated with an IFN-γ gene signature, partly regulated through altered H3 acetylation. Our results could have broader implications for understanding nuances of anti-tumor immune responses in BRCA-null tumors.

## Methods

### Materials

Entinostat, trichostatin A, and etoposide were from Sigma-Aldrich (St. Louis, MO), interferon (IFN) α-1a from Cell Signaling Inc. (Danvers, MA, Cat. No. 8927), and IFN-γ from Gibco (Waltham, MA, Cat. No. PHC4033). Guadecitabine was provided by Astex Pharmaceuticals Inc. (Pleasanton, CA), and GSK126 was purchased from Xcess Biosciences Inc. (San Diego, CA).

### Cells

BRCA1-mutated (BRCA1-null) OC cells UWB1.289, UWB1.289 cells transduced with the BRCA1 gene, and the BRCA1-null breast cancer cell line HCC1937 were obtained from the American Type Culture Collection (ATCC, Manassas, VA). HCC1937 cells with the BRCA1 gene restored using transduction techniques were kindly provided by Dr. E. Swisher (University of Washington, Seattle). UWB1.289 cells were transfected with pcDNA3.1 vector to generate BRCA1-null-vector cells. BRCA1-mutated and BRCA1-normal (wild type) primary mammary epithelial cell lines immortalized by human telomerase gene (hTERT) were obtained as previously described.^50^ The immortalized primary mammary epithelial cell lines were maintained in low glucose Dulbecco’s modified Eagle’s medium (and F12 media (1:3 ratio) supplemented with 5% fetal bovine serum (FBS), 5 μg/mL human insulin, 0.4 μg/mL hydrocortisone, 20 ng/mL EGF, and 1% penicillin–streptomycin solution. UWB1.289 cells were cultured in media containing 1:1 MEBM (Lonza, Elburn, IL, Cat. No. CC-3151) and RPMI 1640 (ATCC, Cat. No. 30-2001) supplemented with 3% FBS, 1% penicillin–streptomycin solution, and MEGM Single-Quots (Lonza, Cat. No. CC-4136). HCC1937 cells were maintained in RPMI 1640 supplemented with 10% FBS and 1% penicillin–streptomycin solution. All primary cells and cell lines were cultured at 37 °C, and 5% CO_2_. UWB1.289 (BRCA1-null), UWB1.289+ BRCA1, and HCC1937 (BRCA1-null) cells lines were obtained authenticated and mycoplasma-free from the ATCC in 2017. Cell lines are tested for mycoplasma contamination in our laboratory, or at IDEXX Bioresearch (Columbia, MO) approximately every 2 years.

### Chromosome immunoprecipitation (ChIP) and ChIP-sequencing (ChIP-seq)

Chromatin was prepared using a kit (#17-295, Millipore, Billerica, MA) following the manufacturer’s protocol. Briefly, chromatin was cross-linked with 1% formaldehyde at 37 °C for 10 min. Cells were treated with SDS lysis buffer and then sonicated (5 pulses of 10 s each at 30-s intervals) using a Fisher Scientific Model 100 sonicator set at 3. Protein–chromatin complexes were immunoprecipitated with ChIP-validated antibodies against H3K9ac (polyclonal, Cat. No. ab4441 used at 1/40; Abcam, Cambridge, MA), H3K27ac (polyclonal, Cat. No. ab4729 used at 1/50; Abcam), STAT1 (Cat. No. sc-346 used at 1/50 dilution; Santa Cruz Biotechnologies, Dallas, TX), or IgG (negative control). Immunoprecipitated complexes were separated with salmon sperm DNA/protein A agarose slurry, followed by chromatin elution, cross-link reversal, and DNA purification (QIAquick PCR purification kit; Qiagen, Germantown, MD). STAT1 localization to regions of the *CXCL10*, *CXCL11*, and *IFI16* promoters containing a gamma interferon activation site (GAS) consensus sequence (TTCN_2-4_GAA) was determined by qPCR using primers flanking the GAS-containing regions (Supplementary Table 1). DNA libraries for H3K9ac and H3K27ac ChIP-seq were prepared and sequenced at the University of Chicago Genomics Facility, Knapp Center for Biomedical Discovery (Chicago, IL).

### RNA-sequencing (RNA-seq)

Total RNA was extracted with the RNA Stat-60 reagent (Tel-Test, Inc., Friendswood, TX). Poly(A) mRNA was isolated using a NEBNext Poly(A) mRNA Magnetic Isolation Module (#7490 S, New England Biolabs Inc., Ipswich, MA) following the manufacturer’s protocols. cDNA libraries were prepared with a NEBNext® mRNA Library Prep Master Mix Set for Illumina (#E6110S; New England Biolabs Inc.), as previously described,^51^ and sequenced at the University of Chicago Genomics Facility, Knapp Center for Biomedical Discovery (Chicago, IL) using a HiSeq instrument (Illumina, Wheeling, IL). Data were deposited in Geo (Accession Number GSE131142).

### Real-time quantitative RT-PCR (qRT-PCR)

An iScript cDNA Synthesis kit (Bio-Rad Laboratories, Hercules, CA) was used for reverse transcription, and iTaq Universal SYBR Green Supermix (Bio-Rad) was used for quantitative PCR (qPCR) amplification of cDNAs following the manufacturer’s procedures. Relative changes in amounts of mRNAs were calculated by the 2^−ΔΔCT^ method using GAPDH for normalization, as previously described.^52^ All experiments were repeated independently at least three times. Regular PCR was performed using GoTaq Green master mix (Promega, Madison, WI). PCR products were resolved by agarose gel electrophoresis and digital pictures of gels were obtained using an image analyzer (ImageQuant LAS 4000; GE Healthcare Life Sciences, Piscataway, NJ). The sequences of primers used for qRT-PCR are presented in Supplementary Table 1. Human Interferons and Receptors RT² Profiler PCR arrays were purchased from Qiagen (Cat. No. PAHS-064Z) and used with an ABI Prism 7900 HT Real-Time PCR system (Applied Biosystems, Foster City, CA), as previously described.^52^

### Western blotting

Proteins were resolved by SDS-PAGE and electroblotted onto polyvinylidene difluoride membranes (Millipore). Membranes were blocked and then probed with antibodies against the proteins of interest. Antibodies against H3K9ac (polyclonal, Cat. No. ab4441, used at 1/500), H3K27ac (polyclonal, Cat. No. ab4729, used at 1/1000), and histone H3 (polyclonal, Cat. No. ab1791, used at 1/1000) were from Abcam Inc.; anti-STAT1 (Cat. No. sc-346, clone E-23, lot #K1115, used at 1/200) and anti-BRCA1 (Cat. No. sc-6954, clone D-9, used at 1/250) were from Santa Cruz Biotechnologies; anti-phospho-STAT1 (Tyr701) (Cat. No. 7649, clone DA47, Lot 5, used at 1/1000), anti-HDAC1 (Cat. No. 5356, clone 10E2, used at 1/1000), and anti-phospho-H2AX (γH2AX, Cat. No. 9718, clone 20E3, used at 1/1000) were from Cell Signaling Technology; anti-β-actin (Cat. No. A1978, clone AC-15, used at 1/2000) was from Sigma-Aldrich; and anti-GAPDH (Cat. No. H86504M, clone B2534M, lot # 22012014, used at 1/10,000) was from Meridian Life Sciences, Inc. (Memphis, TN). Membranes were incubated with HRP-conjugated secondary antibody, followed by detection of antigen–antibody complexes using an enhanced chemiluminescent substrate (SuperSignal West Pico PLUS; Thermo Scientific, Waltham, MA) and a luminescent image analyzer (ImageQuant LAS 4000; GE Healthcare Life Sciences, Piscataway, NJ). Detection of additional proteins on the blots (e.g. total histone H3, Fig. 1; or STAT1 and GAPDH, Fig. 3) was performed applying the above procedure after treatment with Restore Western Blot Stripping Buffer (Thermo Scientific). All blots derived from the same experiment and were processed in parallel. Densitometric analysis of protein bands used ImageJ 1.48 software (https://imagej.nih.gov/ij). Images of blots were cropped to show the proteins of interest. Whole images of representative blots are presented in Supplementary Figs 10–13.

### Histone deacetylase activity

Cell lysates were obtained using RIPA buffer. Nuclear proteins were extracted with an EpiQuik Nuclear Extraction kit (EpigenTek, Farmingdale, NY) following the manufacturer’s instructions. Total HDAC activity in cell lysates or nuclear extracts was determined using a HDAC Activity Colorimetric Assay kit (EpigenTek). Absorbance was measured by an EL800 microplate reader controlled by Gen5 version 2.09 software (BioTek Instruments, Inc., Winooski, VT). HDAC activity was expressed as OD/mg of protein.

### Cell proliferation assay

The Cell Counting Kit-8 assay (CCK8, Dojindo Molecular Technologies, Inc., Rockville, MD) was used to measure cell proliferation following the manufacturer’s protocol. Absorbance at 450 nm was determined by an EL800 microplate reader and Gen5 version 2.09 software (BioTek Instruments, Inc., Winooski, VT).

### Immunofluorescence

Cells were grown in eight-well culture slides (Millicell EZ slide, Millipore) at the density of 20,000 per well. Cells were fixed with 4% paraformaldehyde for 30 min, and then blocked and permeabilized for 60 min with PBS containing 5% normal goat serum and 0.3% Triton X-100. Cells were incubated with primary antibody (anti-phospho-H2AX, Cat. No. 9718, clone 20E3, Cell Signaling, used at 1/200 dilution) overnight at 4 °C, and then with Alexa Fluor 488-labeled anti-rabbit IgG (Cat. No. A11008 used at 1/500, Molecular Probes, Eugene, Texas). Slides were coverslipped using Vectashield mounting medium with DAPI (Vector Laboratories, Burlingame, CA). Digital pictures of stained cells were obtained using an AxioCam HRC camera attached to an AxioVert200 inverted microscope and Axiovision release 4.5 software (Carl Zeiss Microscopy, White Plains, NY).

### Cell transfection and transduction

Stable knock-down of *STAT1* was performed by RNA interference using small hairpin RNAs (shRNAs). Lentiviral particles containing shRNAs targeting STAT1 or control shRNAs were obtained from Sigma-Aldrich (MISSION TRCN0000280024 and TRCN0000004264). Transduced cells were selected by antibiotic resistance (puromycin, 2 μg/mL). The pcDNA3.1 vector (Addgene, Watertown, MA) was transfected into UWB1.289 cells using TurboFectin 8 reagent (OriGene, Austin TX), and stably transfected cells (named BRCA1-null-vector) were selected with G418 (600 μg/mL).

### Statistical analysis

RNAseq data were aligned to assembly HG19 with Tophat 2.^53^ Quality control and gene quantification were conducted with NGSUtils,^54^ and differential expression analysis was done with Bioconductor edgeR package in R. Mutation data and clinical data for HGSOC were downloaded from the TCGA portal (version 02/11/2016). The gene expression data were obtained on a custom gene expression microarray Agilent G4502A, and normalized level 3 data were downloaded. Four exome sequence datasets (BCM SOLiD; BI IlluminaGA; WUSM IlluminaGA; WUSM IlluminaHiSeq) were downloaded from TCGA portal,^1^ and 19 samples were identified as carriers of BRCA1 mutations in at least one of the exome datasets. Samples were then classified as BRCA1-deficient or BRCA1-normal controls based on gene expression level of BRCA1 and BRCA1 mutations profile in the exome regions. From a total of 589 samples, 75 samples were considered BRCA-deficient, including 56 samples with expression levels of BRCA1 lower than the 10% quantile and 19 samples containing somatic mutations in the BRCA1 exome regions. Three hundred and thirty-one specimens were considered BRCA1-normal as BRCA1 expression levels exceeded the first quartile and no somatic mutations were observed in the exonic regions of BRCA1 or BRCA2 genes. Samples not included within one of these two groups were removed from further analyses. Analysis of differential gene expression between the BRCA1-deficient and BRCA1-normal groups was conducted with Student’s *t*-test in statistical environment R, and Benjamini & Hochberg False Discovery Rate (FDR) was used to adjust for multiple comparisons. FDR < 0.05 was considered significant.

ChIP-sequencing reads were aligned to the human reference genome (GRCh38) using Bowtie version 1.2.2 with options-best -m1.^55^ Subsequent analyses including peak calling, peak merging, and differential peak analysis were performed with HOMER version 4.10 (http://homer.ucsd.edu/homer/) as described below. Alignment files were first converted to HOMER compatible tag directories using command makeTagDirectory with option -tbp 1 to ignore duplicate reads. Peaks were called relative to their respective inputs using HOMER’s findPeaks program with default settings and option -style histone. Consensus peak lists for each histone modification ChIP-seq were created using HOMER’s mergePeaks command. These merged peaks sets were subsequently analyzed for differential H3K27Ac or H3K9Ac tag density in BRCA1-null versus BRCA1+ cells using HOMER’s getDifferentialPeaks command with fold change cutoffs of two-fold and Poisson enrichment *P* value < 0.0001 (default). Location of H3K27Ac or H3K9Ac peaks relative to genomic regions of interest (i.e. promoter, intron, exon, TSS) and distance to nearset transcription start site was determined using HOMER’s annotatePeaks command. Heatmap visualizations and read per genome coverage normalized bigwig files were generated in deepTools2 version 3.1; bigwig files were visualized using IGV (http://broadinstitute.org/igv).

Co-expression among the genes *CXCL10*, *CXCL11*, *IFI16*, and *IFNG* was estimated by linear regression using the R software, and a *P* value < 0.05 was considered statistically significant. We transformed the dependent variables in our regression models and fit the regression with transformed dependent variables. We conducted a cubic root transformation to the expression level of gene IFNG, and fitted the regression model on the transformed *IFNG* on *CXCL10, CXCL11*, and *IFI16*. We verified the homoscedasticity assumption with Breusch-Pagan (BP) test for the new regression.

The Kaplan–Meier method was used to estimate the probabilities for overall survival and disease-free survival, and the univariate Cox Proportional Hazard regression model was used to compare survival differences between subgroups. A summary of the information pertaining to Reporting Recommendations for Tumor Marker Prognostic Studies (REMARK) is presented in Supplementary Table 2. Tumor Immune Estimation Resource (TIMER) was used to estimate the proportion of immune cells subsets infiltration in BRCA1-deficient and BRCA1-normal tumor subgroups.^34^ Gene expression data obtained by RT-PCR, and cell survival, were analyzed using the Student’s *t*-test (two-sided) available on Excel 2013 for Microsoft Windows. Measurements were taken from distinct samples and averaged within experimental groups. RT-PCR measurements per sample were the average of three determinations.

## Supplementary information

Supplemental Material

## Data Availability

RNA-seq and ChIP-seq datasets generated in this study are available at the Gene Expression Omnibus (GEO) database, National Center for Biotechnology Information (Super-Series Accession # GSE131142, https://www.ncbi.nlm.nih.gov/geo/query/acc.cgi?acc = GSE131142). The figures associated to these data are Fig. 1d–f, Fig. 5a–f, and Supplementary Fig. 4.
