# Supplementary material for: Interferon-γ signaling is associated with BRCA1 loss-of-function mutations in high grade serous ovarian cancer
Source: NPJ Precis Oncol. 2019 Dec 6;3:32. doi: 10.1038/s41698-019-0103-4 (PMC6897992; doi:10.1038/s41698-019-0103-4)
Supplement: Supplementary file 1 — Supplemental Material [file 41698_2019_103_MOESM1_ESM.pdf]

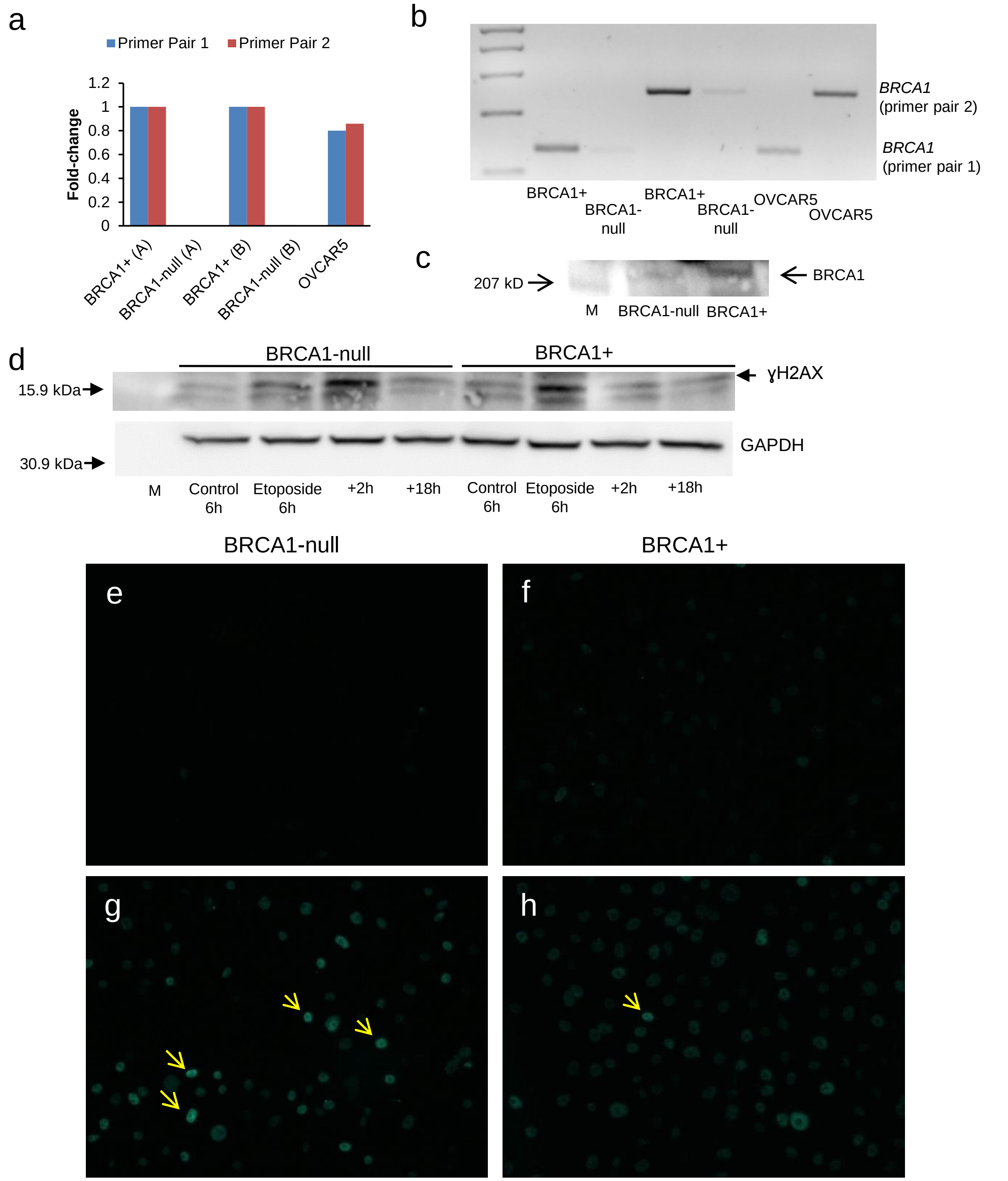

Supplementary Figure 1. Demonstrations of the 2594delC mutation in the BRCA1 gene, and ds DNA break repair in BRCA1+ and BRCA1-mutated (BRCA1-null) UWB1.289 ovarian cancer cells. **a, b.** Amplification of a *BRCA1* mRNA fragment expected to contain the 2594delC mutation in BRCA1+, BRCA1-null (samples A and B were obtained at different times), and OVCAR5 (positive control) cells by real-time (**a**) and regular RT-PCR (**b**). The 3'-end of the forward primer was designed to bind the 2594C nucleotide of the *BRCA1* transcript (absent in BRCA1-null), and was paired with 2 different reverse primers. **c.** Western of BRCA1 in BRCA1-null and BRCA1+ cell lines using an antibody targeting the C-terminus. M, protein markers. **d**. Western of ɣH2AX at the end of etoposide treatment (25 μM for 6h), and at 2h and 18h after etoposide wash-off. M, protein markers. **e, f, g, h.** Immunofluorescence of ɣH2AX 2h after DMSO (**e, f**) or etoposide (25 μM, 6h, **g, h**) treatment. Arrows point at some cells showing relatively high levels of ɣH2AX staining.

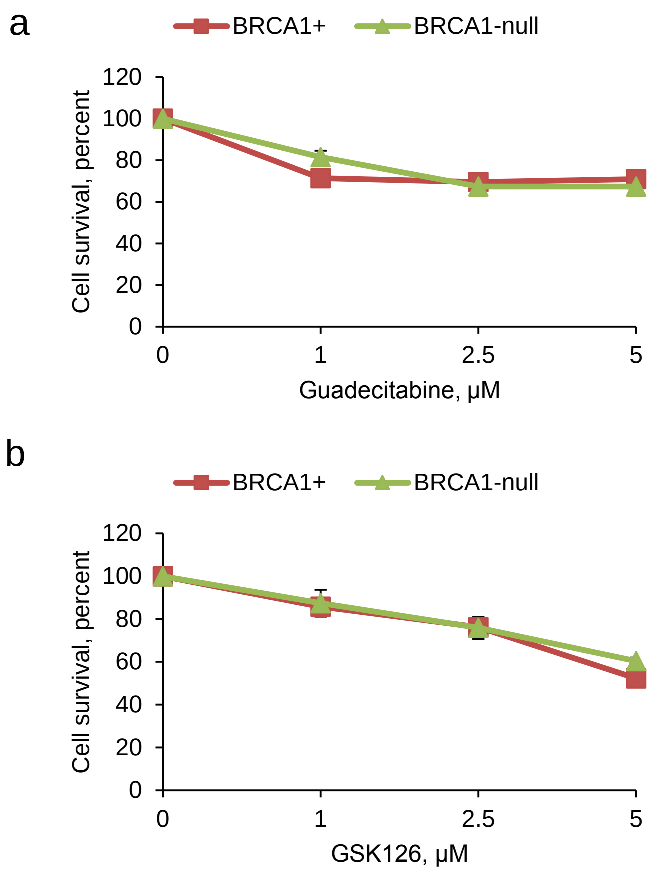

Supplementary Figure 2. A *BRCA1* loss-of-function mutation does not alter effects of DNA methylation or H3K27 methylation inhibitors on survival of ovarian cancer (OC) cells. a, b. Survival measured by the CCK8 assay of *BRCA1*-mutated (BRCA1-null) and BRCA1+ UWB1.289 ovarian cancer cells treated with the DNA methylation inhibitor guadecitabine **(a)**, or EZH2 (H3K27 methyltransferase) inhibitor GSK126 **(b)** for 72 hours**.** Values represent means ± s.e.m. (n=3 replicates).

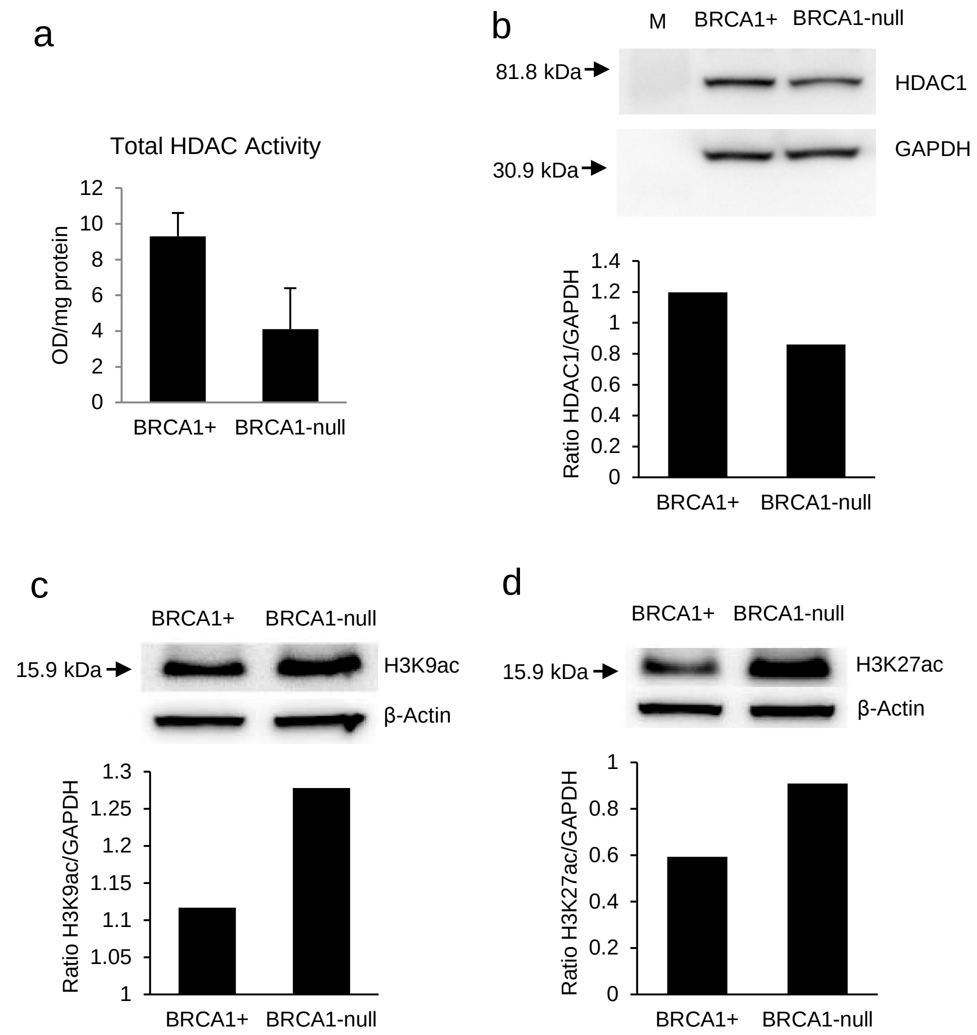

Supplementary Figure 3. HDAC activity, HDAC1 protein levels, and histone H3 acetylation in BRCA1-mutated (BRCA1-null) and BRCA1+ ovarian cancer cells. a. Total HDAC activity (means ± s.e.m., n=2 replicates). Western blot and results of densitometric analysis of protein bands for HDAC1 (**b**), H3K9ac (**c**), and H3K27ac (**d**). M, protein markers

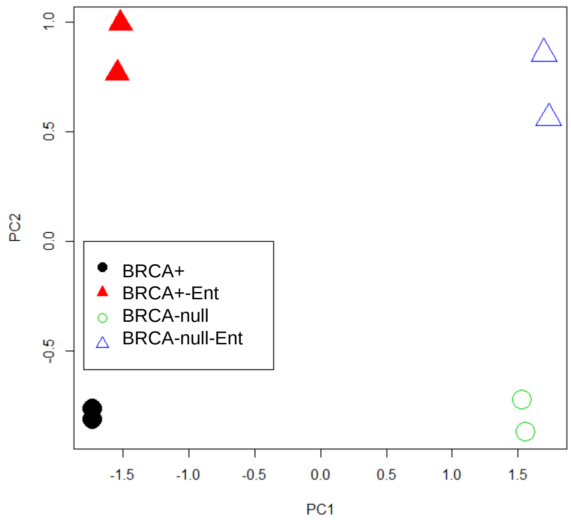

Supplementary Figure 4. Principal component analysis of transcriptomic data obtained by RNAseq in BRCA1+ and BRCA1-mutated (BRCA1-null) UWB1.289 ovarian cancer cells treated or not treated with the histone deacetylase (HDAC) inhibitor entinostat (Ent, 0.5 μM).

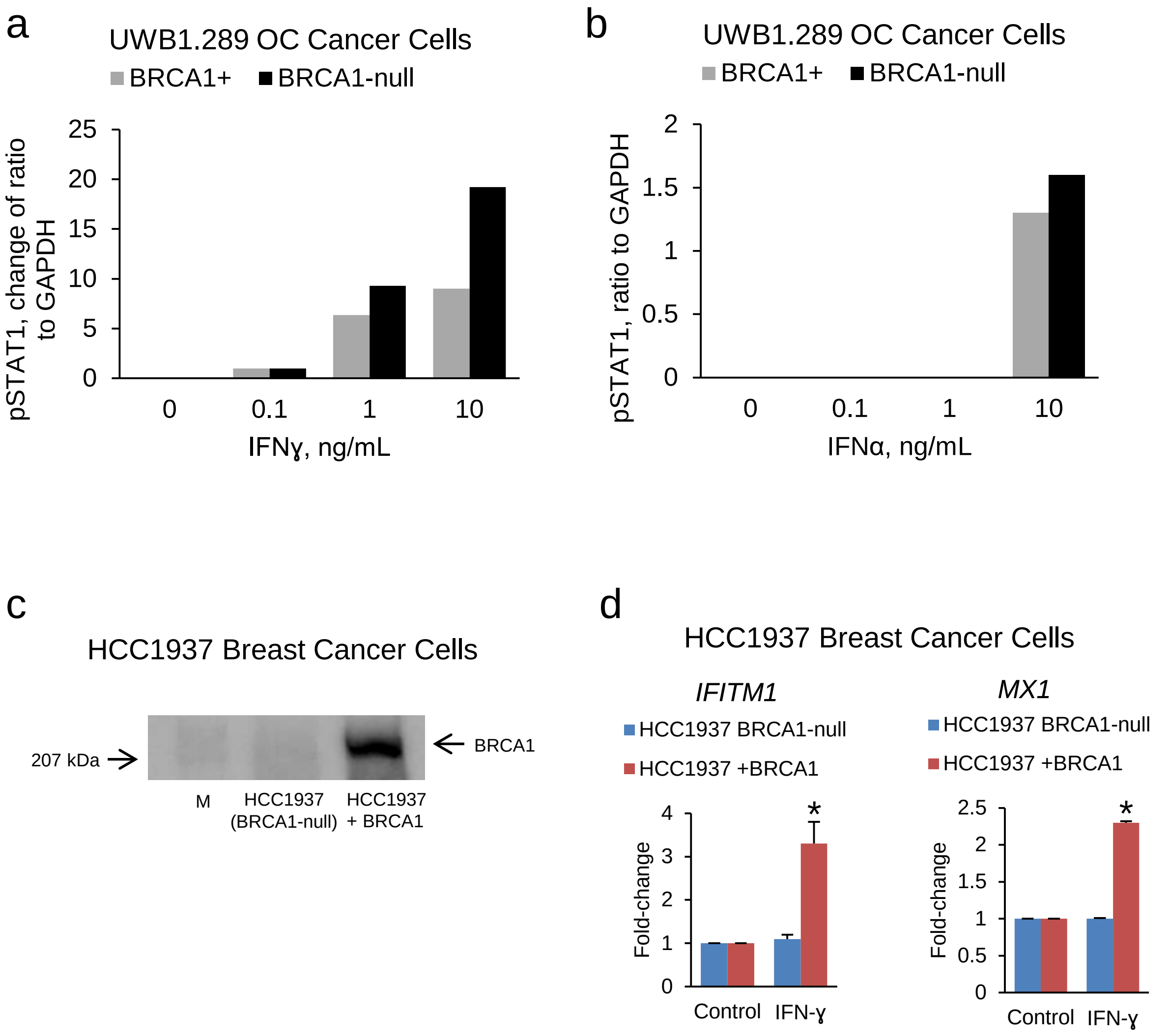

Supplementary Figure 5. **a**. Densitometric analysis of pSTAT1 bands presented in Fig. 3b. **b**. Densitometric analysis of pSTAT1 bands presented in Fig. 3e **c**. Western of BRCA1 in BRCA1-mutated HCC1937 (BRCA1-null) and HCC1937+BRCA1 breast cancer cell line. M, protein markers. **d**. Levels (means ± s.e.m.) of *IFITM1* and *MX1* mRNAs measured by qRT-PCR in HCC1937 (BRCA1-null) and in HCC1937+BRCA1 breast cancer cells treated with IFN-γ (10 ng/mL) for 24 hours. **P*<0.05 (t-test) relative to control, n=3 replicates.

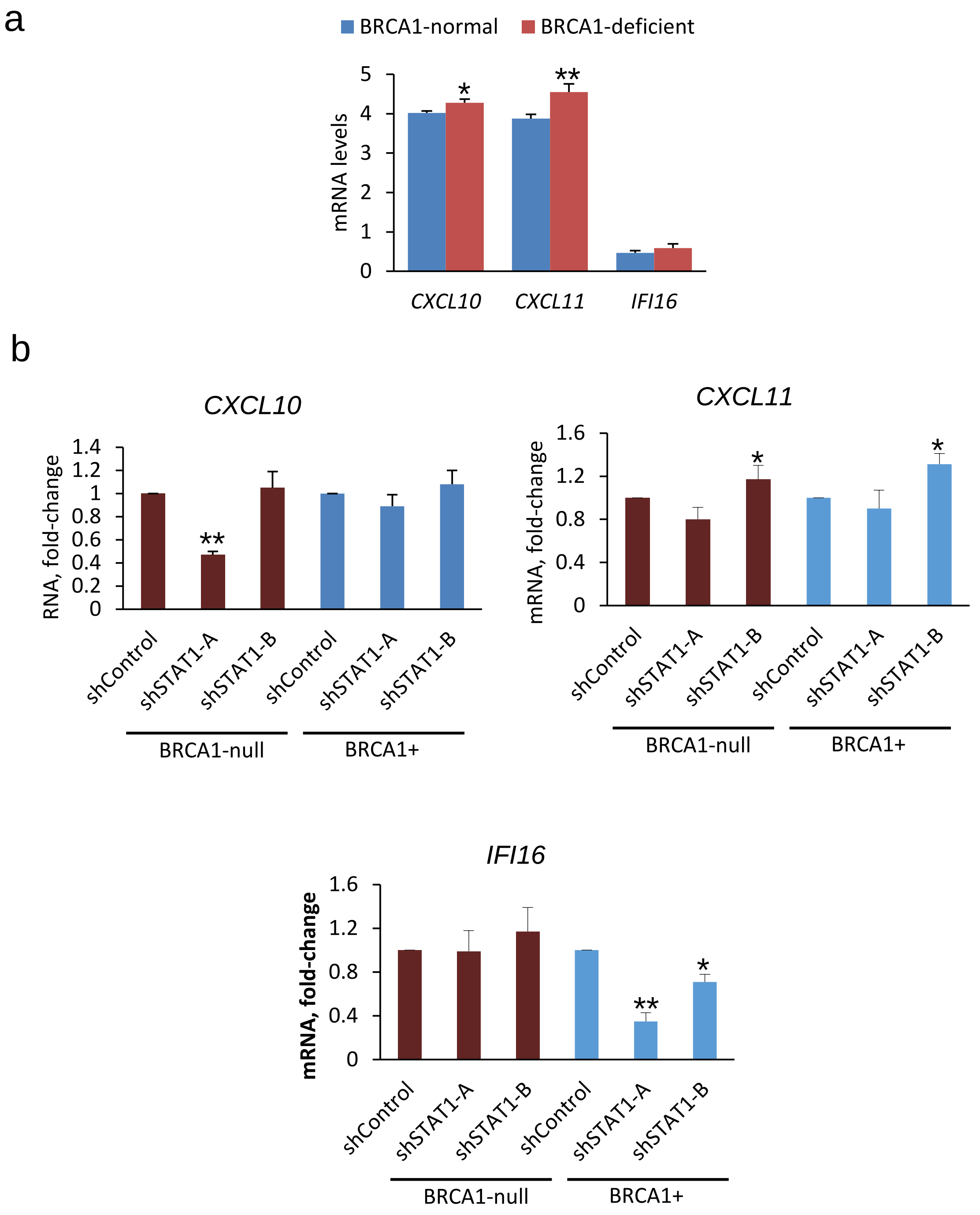

Supplementary Figure 6. mRNA levels (means ± s.em.) of the IFN-γ-regulated genes *CXCL10*, *CXCL11*, and *IFI16* in BRCA1-normal (n=330) and BRCA1-deficient (n=75) ovarian cancer tumors from the TCGA (**a**), and in BRCA1-null and BRCA1+ UWB1.289 ovarian cancer cells transduced with non-targeting shRNA (shControl) or shRNAs targeting *STAT1* (shSTAT1-A and –B). Values are means ± s.em., n=3 replicates (**b**). ** *P*<0.01, **P*<0.05, t-test.

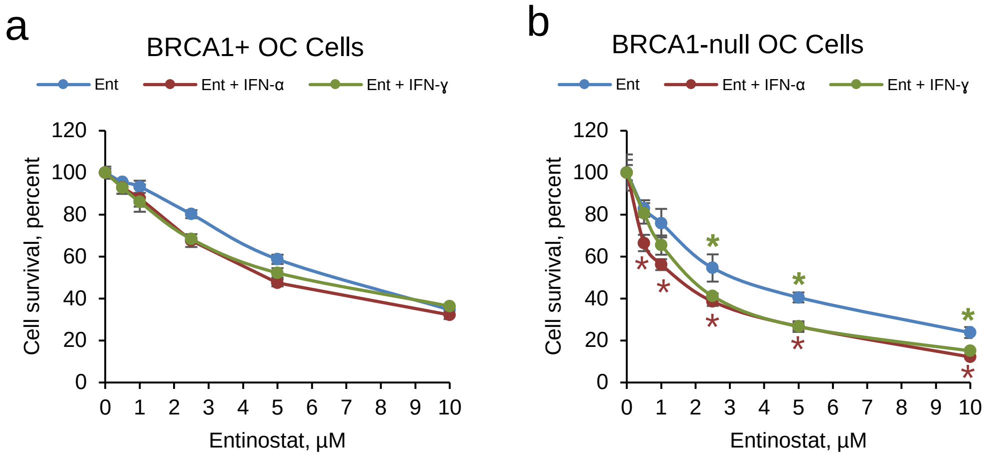

Supplementary Figure 7. Responses of ovarian cancer (OC) cells to IFN-ɣ depended on BRCA1 mutational status. a, b. Survival of BRCA1+ (a) and BRCA1-mutated (BRCA1-null) (b) UWB1.289 OC cells treated with the HDAC inhibitor entinostat (Ent), or Ent concomitantly with IFN-α (10 ng/mL) or IFN-ɣ (10 ng/mL) for 72 hours. *$P<0.05$ (t-test) relative to Ent alone at the indicated doses. Values are means ± s.e.m., n=4 replicates.

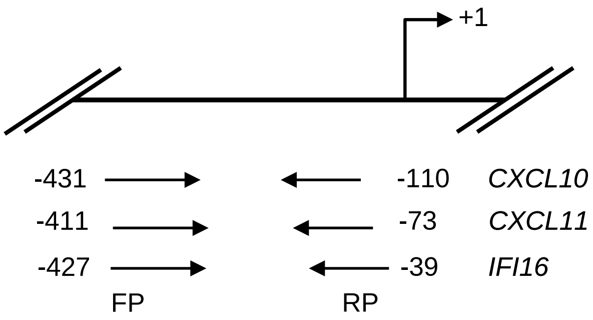

Supplementary Figure 8. 5’ binding sites relative to TSS (+1) for primers used for PCR amplification of *CXCL10*, *CXCL11*, and *IFI16* promoter fragments after H3K9ac ChIP in the BRCA1-mutated UWB1.289 ovarian cancer cell line transduced with empty vector or vector containing a BRCA1 sequence (data shown in Fig. 5G). FP: forward primer, RP: reverse primer.

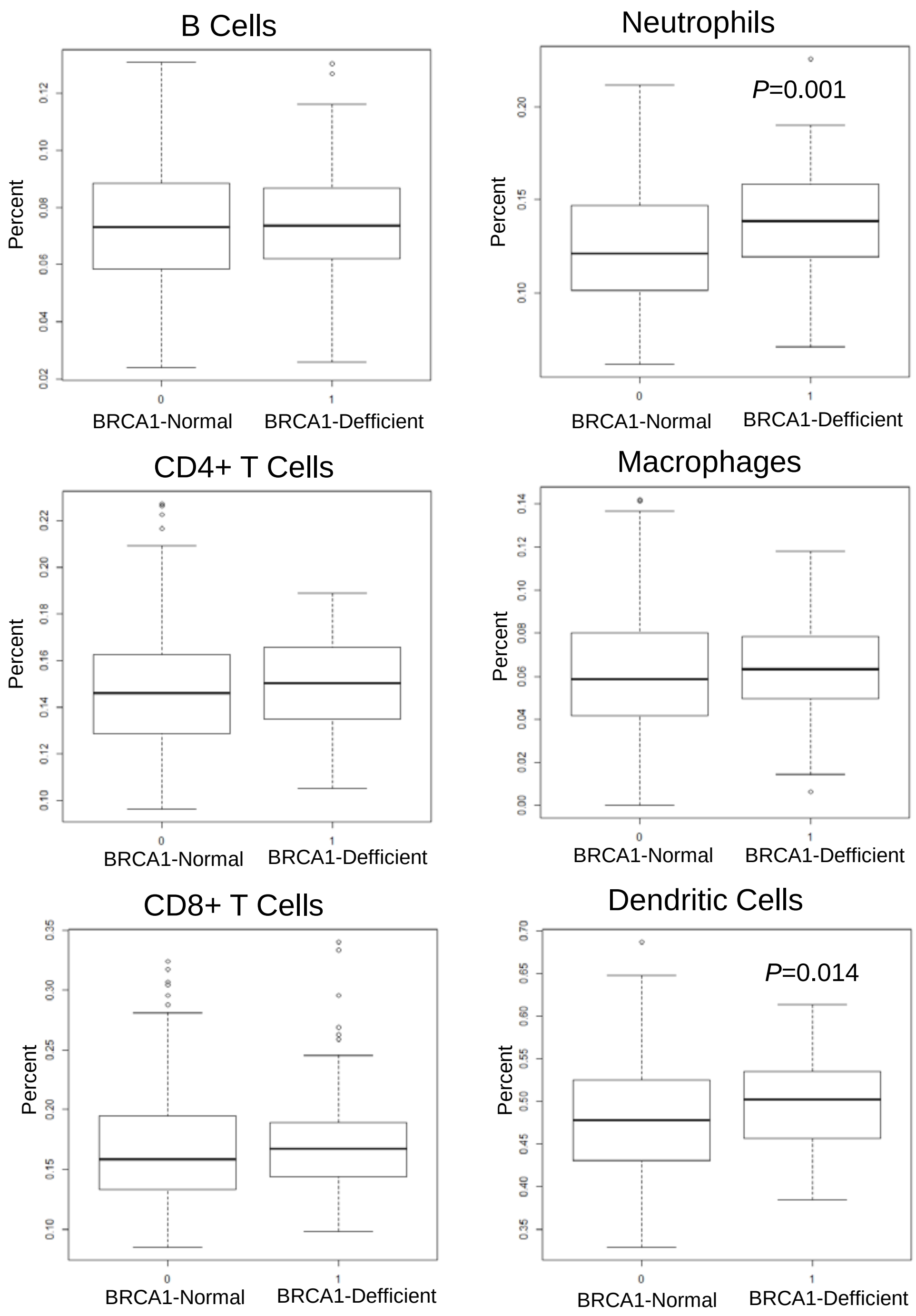

Supplementary Figure 9. Infiltrating immune cells in ovarian cancer tumors (TCGA). Proportions of infiltrating immune cells in BRCA1-normal and BRCA1-defficient ovarian cancer tumors (TCGA) determined using the Tumor Immune Information Resource (TIMER). Box plots depict median and 25-75% quartiles, whiskers represent data range. Shown are t-test *P*-values.

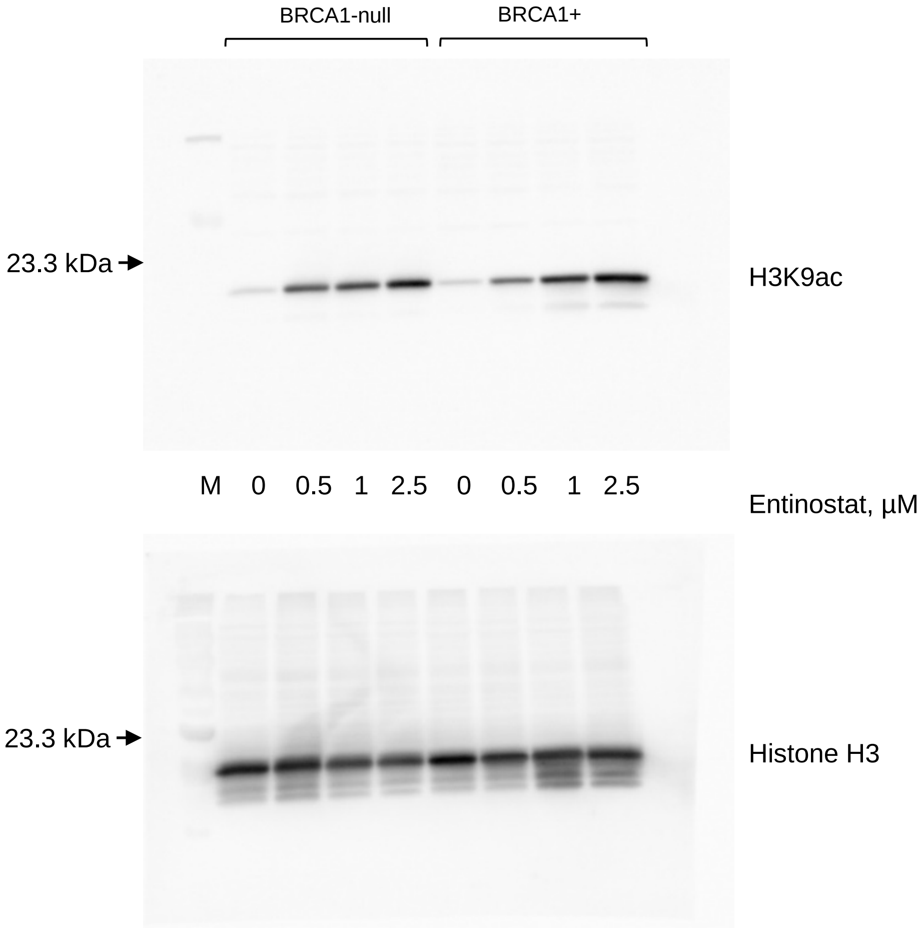

Supplementary Fig 10. Images of the whole blots from which the cropped images presented in Figure 1B were obtained. M, protein markers.

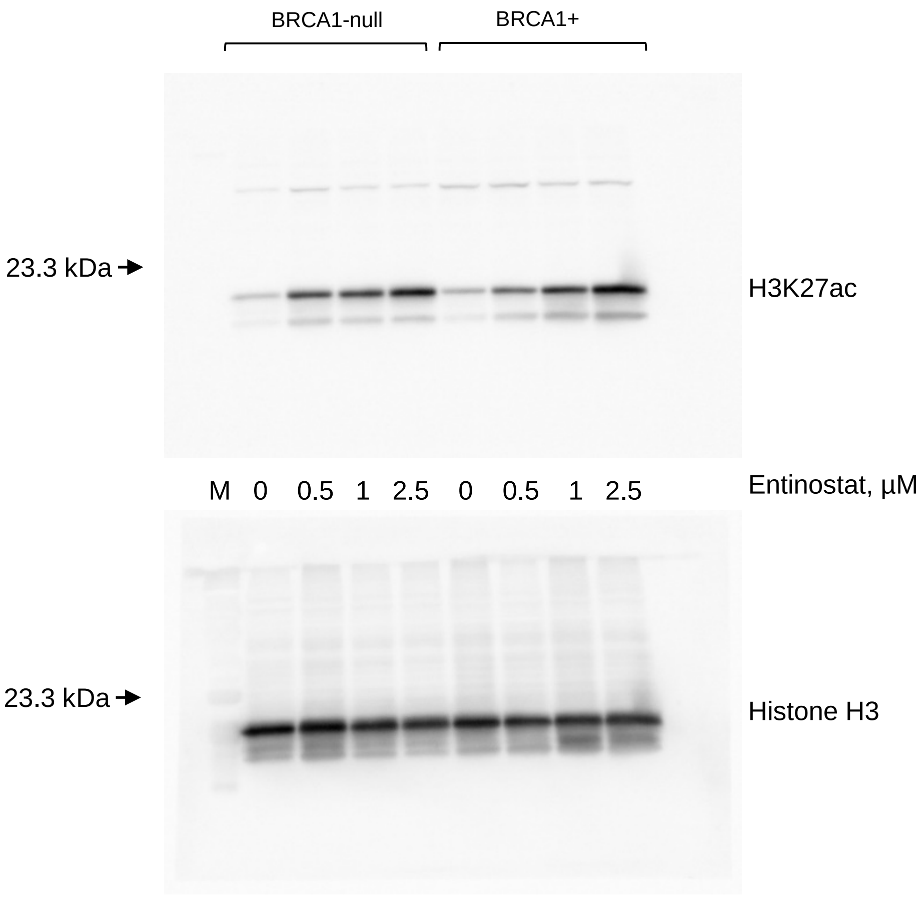

Supplementary Figure 11. Images of the whole blots from which the cropped images presented in Figure 1C were obtained. M, protein markers.

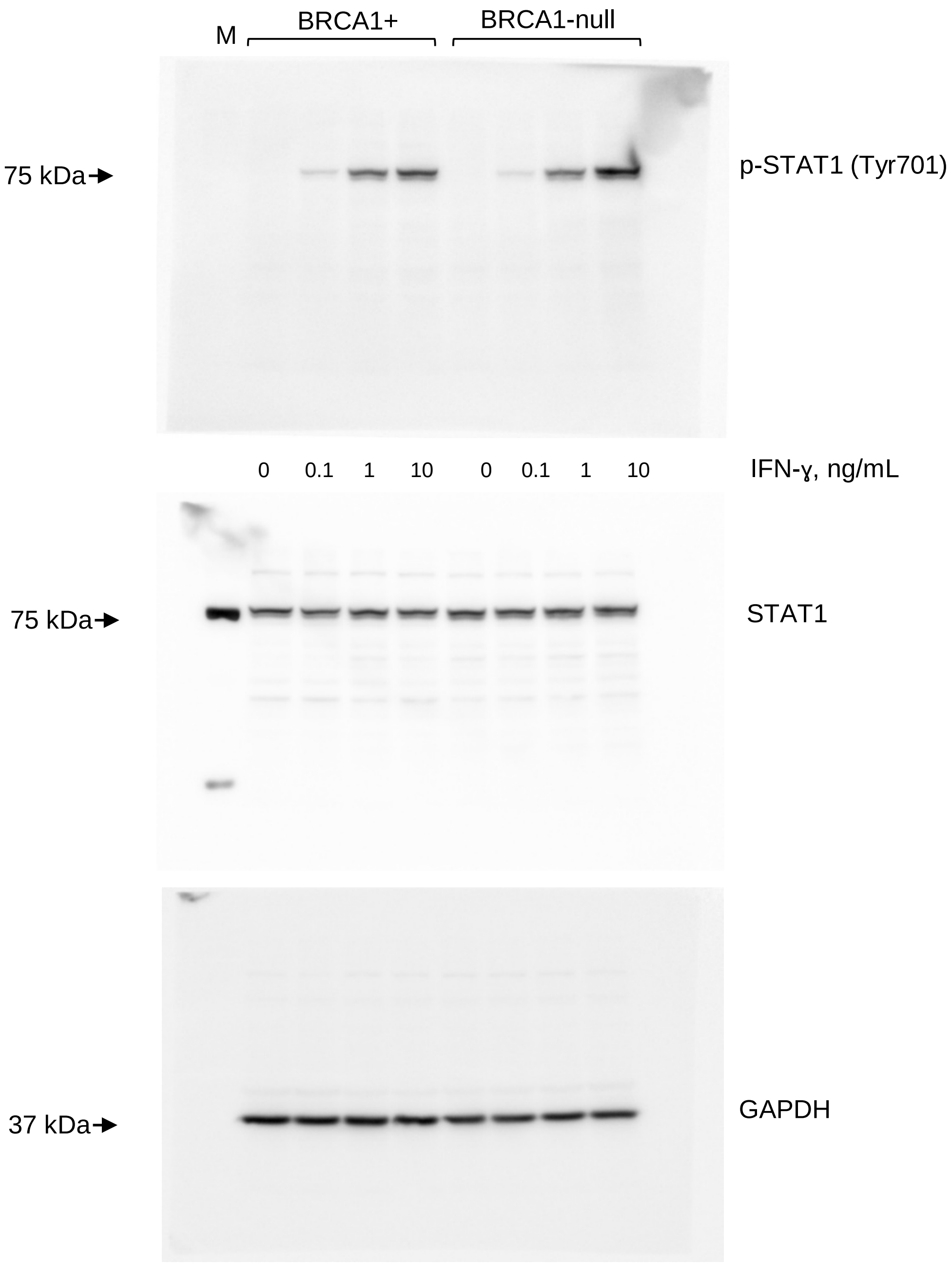

Supplementary Figure 12. Images of the whole blots from which the cropped images presented in Figure 3B were obtained. M, protein markers.

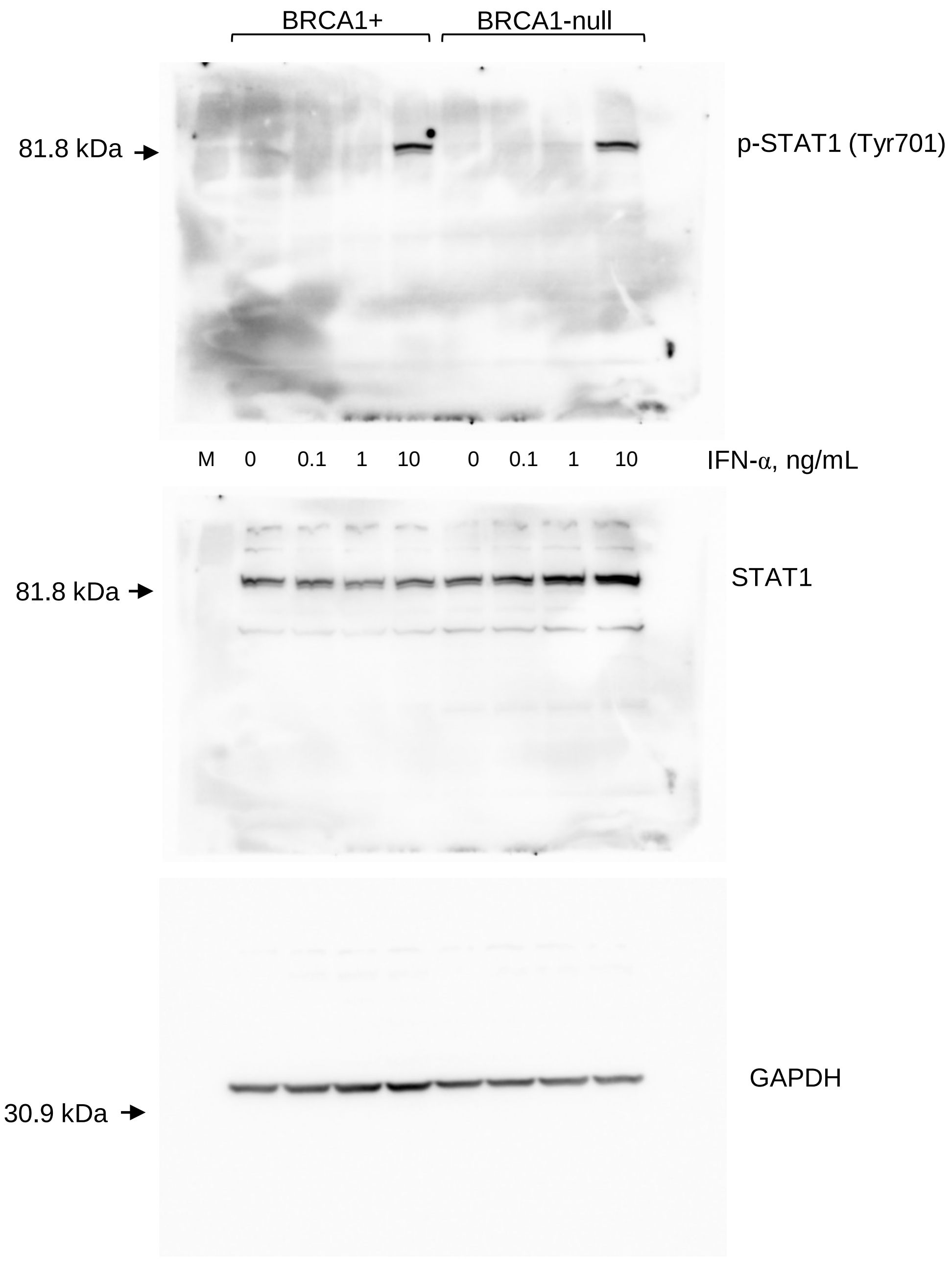

Supplementary Figure 13. Images of the whole blots from which the cropped images presented in Figure 3E were obtained. M, protein markers.

Supplementary Table 1. Sequences of the primers used for qRT-PCR and ChIP.

Gene	Forward (5' to 3')	Reverse (5' to 3')
CXCL10	AAAGCAGTTAGCAAGGAAAG	TCAFFGGTCACCTTTTAGTG
CXCL11	CTACAGTTGTTCAAGGCTTC	CACTTTCACTGCTTTTACCC
IFI16	TTAACCACGTTGAAACCAAG	TCGGAAGACTTCATTCTCAG
IFITM1	CTACTCCGTGAAGTCTAGG	ATGAGGATGCCCAGAATC
MX1	CTGACTCTAATAGACCTTCCTG	GATCTTATACCCAATGTCAGC
GAPDH	AGCCACATCGCTCAGACAC	GCCCAATACGACCAAATCC
STAT1	ACCCAATCCAGATGTCTATG	GAGCCTGATTAAATCTCTGG
BRCA1	GACTGTTTATAGCTGTTGGAAG	TTTTGGAAGTGTTTGCTACC
BRCA1(2594delC)	TCCAAAGATAATAGAAATGAC	AAACCTTGAATGTATTCTGC (Rev. 1)
BRCA1(2594delC)	TCCAAAGATAATAGAAATGAC	ATTCAAAAGTGACTTTTGGAC (Rev. 2)
CXCL10 (ChIP STAT1)	CCCAAATGAGCAATGTTTTCCCT	GGTCAAAGATCTGGAACTGGC
CXCL11 (ChIP STAT1)	ATCAGAATTCCGCTGCCCAA	GTGAAAGCACTGTGAAAACCCA
IFI16 (ChIP STAT1 GAS1)	TACAACATCCTGCGGTTCCG	GGAACAGTACTCACCTCACTGG
IFI16 (ChIP STAT1 GAS2)	CCAAGATCTCCTCCTGCCAC	ACCCAGCAGACTTACATGTGA
CXCL10 (ChIP H3K9ac)	GGCACAACTTGCTGTTACCAA	GGGAAGTCCCATGTTGCAGA
CXCL11 (ChIP H3K9ac)	GGACTGAAAAGCAAAAGTGAGAT	TCCCTCTTTGAGTCATGCACC
IFI16 (ChIP H3K9ac)	GAGGGGAGAAGAAGCCATGTTG	GAGACTCCTCCCACCAGTGTT

Supplementary Table 2. Checklist containing information pertinent to Reporting Recommendations for Tumor Marker Prognostic Studies (REMARK).

**Introduction**	
1	We investigated the relationship between an IFN-γ signature and clinical outcomes of high grade serous ovarian cancer patients from the TCGA database. The specific objective was to determine whether mRNA expression levels of the IFN-γ-regulated genes *CXCL10*, *CXCL11*, and *IFI16* in patient tumors was associated with patient survival.
**Materials and Methods**	
Patients	
2	We studied high grades serous ovarian cancer tumors from patients included in the TCGA database (version 02/11/2016). Four exome sequence datasets (BCM SOLiD; BI IlluminaGA; WUSM IlluminaGA; WUSM IlluminaHiSeq) corresponding to 589 samples (patients), mutation and clinical data were downloaded from the TCGA portal.
3	Data used for analysis were obtained before patients received chemotherapy.
**Specimen characteristics**	
4	Tumor samples were obtained at the time of surgery and processed for RNA extraction and analysis of gene expression using RNA microarrays.
**Assay Methods**	
5	Transcript levels were determined using the Agilent G4502A microarray.
Study Design	
6	Samples were classified as BRCA1-deficient or BRCA1-normal based on gene expression levels of BRCA1 and BRCA1 mutation profiles in the exome regions. 75 samples were considered BRCA deficient (56 with BRCA1 mRNA lower than the 10% quantile and 19 containing BRCA1 somatic mutations). 331 specimens were considered BRCA1-normal as BRCA1 mRNA levels were within the 25% quartile and no somatic mutations were observed in the exomic regions of BRCA1 or BRCA2 genes. Samples not included within one of these two groups were excluded. To determine whether the IFN-γ signature carried prognostic value, OC samples were classified as "high expressing" subgroup, if the expression of the IFN-γ-regulated genes *CXCL10*, *CXCL11*, and *IFI16* was higher than their corresponding median values or as "low expressing" subgroup, if the expression level for the 3 genes was lower than the median value. Seventy three and 84 ovarian tumors were thus classified as "high" and "low" subgroups respectively, and survival differences between these subgroups were determined.
7	Clinical endpoints: Progression-free survival and overall patient survival.
8	Candidate variables: mRNA expression levels of the IFN-γ-regulated genes *CXCL10*, *CXCL11*, and *IFI16*, alone or in combination.
9	Sample size: The study was not designed to detect a specific effect size. We used all samples available in the TCGA database that met the criteria specified in Section 6.
Statistical	

Analyses	
10	The Kaplan-Meier method and the univariate Cox proportional hazard regression model were used to estimate differences in overall survival (OS) and disease-free survival (DFS) between groups.
11	Marker: An IFN-γ signature was detected in BRCA1-defficient samples based on gene expression analysis of IFN-γ-regulated genes *CXCL10*, *CXCL11*, and *IFI16*. Subgroups having high or low relative expression of these genes were formed and associations with patient survival were determined.
**Results**	
Data	
12	Flow of patients through the study: Patients were those included in the TCGA portal.
13	Patient demographics: No patient demographics were collected for the present study.
Analysis and Presentation	
14	Patients having high expression levels of IFN-γ regulated genes *CXCL10*, *CXCL11*, and *IFI16* (elevated IFN-γ signature) had longer overall survival (*P*=0.05, Cox proportional hazard regression) than patients with low expression levels.
15, 16,17,18	A Kaplan-Meier plot and distribution of number of patients at risk (Fig. 6D) shows the relationship of IFN-γ signature levels with overall survival.
Discussion	
19, 20	Included in the Discussion section of the manuscript.

Supplementary Table 3. Unique genes (total of 1738) induced by entinostat in BRCA1+ compared with BRCA1-null OC cells (*P*< 0.01, t-test, FDR<0.05). Gene expression was measured by RNAseq.

PDGFRA	WDR17	NOTCH2NL	KDM3B	ZNF532
SYT5	MMP19	APAF1	CNDP2	RBM19
PRSS35	RND2	ATXN1	SLC25A13	MCM5
CLCNKA	RHOV	MANBA	HSPE1	ELK4
BAI3	CD83	LOC100506990	DEPDC1	NOL6
PURG	RARRES2	ACBD4	QRICH1	SARS2
GLI1	IFIT5	C20orf96	NF2	MID1IP1
S1PR5	LOC102724508	IL27RA	TFDP1	SF1
TMEM151A	NOV	CACHD1	YAP1	ECE2
RASD2	AKAP6	SCD5	PITRM1	MAP3K6
MIR6795	SPATA6	B4GALNT4	ENOSF1	QSOX2
PRSS3	TCEAL3	CTSB	GLE1	MAF1
SNCG	KDELC1	NCEH1	SENP3-EIF4A1	NFXL1
GGT1	ELAVL2	EHHADH	NELFB	ZNF326
SPSB4	SNAP25	LPPR2	PPP1CA	XRCC3
CHST9	BIRC3	FLNC	SF3A1	HUS1
NDNF	GPRC5B	SLC6A15	PSMD7	MRPL36
DLX4	CCDC69	RPL32P3	POLDIP2	VWA9
SPATA18	SYT12	RAPH1	BOD1L1	TCERG1
ENPP3	STEAP4	NTAN1	CDK2AP1	SURF6
DACH1	JAM2	EPB41L5	NSD1	DPH2
KPNA7	KHK	PLAU	MCUR1	MLST8
C1QL4	ENTPD2	CLDN6	SRP68	SMAD1
AQP3	FUT2	SCARB2	TYMS	ZNF202
GPR17	SBK1	CCND3	PSMD6	GEMIN4
TMEM59L	CSPG5	ARL3	B4GALT3	PAGR1
C1orf95	LINC00501	HMGN3	DYNC1LI1	ST20-MTHFS
GALNT16	S1PR2	RASA4	BMS1	NOL8
TEPP	C4orf19	PLD1	PKP2	KDM2B
SCN4B	PTGES	TANGO6	EIF1AX	HIGD2A
SLC44A5	SEC16B	PRKAB2	LIG1	HIVEP1
CNN1	LOC100128885	ZC4H2	SLC3A2	TRAPPC2L
PHOSPHO1	CHST11	GNAL	RNF187	NOP2
UPB1	CPE	IGFBP7-AS1	IRAK1	WWP2
PPM1E	LRRC75B	GPRC5A	RPF1	VPS9D1-AS1
CYP4X1	FAM49A	MITF	AURKA	YDJC
TCEA3	PGPEP1	FGFBP1	NUP88	COBLL1
TMEM204	RHEBL1	LIX1L	NOP9	AKAP8
AK7	VASH2	SLC17A5	PCNA	TARBP2

TIMP4	SLC25A27	DNAH11	TARS	SREK1
FEZF1	TMEM108	MALAT1	TOMM6	PUS7
PPP1R36	NEDD9	VDR	PITPNB	ZADH2
IL22RA1	GKAP1	N4BP3	SIGMAR1	METTL8
RNF150	IFITM2	PORCN	SEMA3A	PSMG4
FAM212A	CSNK1G2-AS1	MAP7D1	TUBA1C	CTBP1-AS2
MFNG	VWDE	CHST7	DDX56	HMGB2
SPTBN4	DCAF12L2	RHPN2	ALG3	GNAQ
SRRM3	MAP1B	JRKL	HERC2	C1orf109
CCDC160	MCF2L2	LEPRE1	CDC20	PMF1
GABRA2	PITX2	SLC9A7	SLC2A4RG	SUV420H1
CNIH2	B4GALT1-AS1	LPGAT1	MORC2	FAM86A
CHD5	C19orf80	TSTD1	UBE2C	ZMYND11
HECW1	TMEM92	TTC9	DAZAP2	AATF
ERVV-2	BOLA3-AS1	THEM4	ATP11C	C9orf41
RNASE4	CRLF1	TNFAIP8L1	ERGIC2	RPAP2
SIX3	LCN2	LPCAT4	RTCB	ZIC1
MB	NEURL1B	NT5DC3	GPS1	NUDT16L1
LMOD1	GPRASP2	RAB3B	PSMG1	PUS1
ANG	MIR1262	KLC3	PRPF19	CTPS1
KCNH8	VASN	MARK4	EIF4A1	POLE4
STX11	PRAF2	GPR56	CLUH	IGHMBP2
SOBP	ISL2	CALCOCO2	SSFA2	MRPS26
UCP1	GSDMB	ITGB6	HNRNPA3	JMJD4
SLC38A4	GALM	DDIT4	TIAL1	TSSC4
LINC00882	UPK3B	TMEM38A	PPIP5K2	PMF1-BGLAP
RGS17	NAP1L2	BRCA1	SHMT1	WASH5P
MAPK8IP2	NEFM	PCDH1	TNPO2	E2F5
CYP1B1	SLC2A10	CSRP2	LSS	BRMS1L
PPP2R2C	ANGPTL4	C11orf49	OSBPL2	MTIF2
TLE6	CRAT	GLTP	PHB	EXOSC4
ARHGAP26-AS1	TMPRSS13	PRSS21	PCNXL3	GPATCH3
TMEM221	METTL7A	FLJ22184	DDX41	SLC6A6
PDGFRL	CASC10	PPM1H	UBR3	DPH1
FIBCD1	PTGS1	TP53BP1	ABCE1	DHCR7
LOC100128164	DEF6	CDKN2B	NOP56	PTPN18
KRT5	LINC00883	NUCB2	PSMB5	FAM35A
RAB39A	SRSF12	FAM129A	TAF1D	TFAP4
CRABP1	PDGFB	BMP1	RNF103-CHMP3	MARVELD2
CLIC6	NT5M	FAM8A1	GNL2	SFXN5
LRRC63	FCGRT	ITGA2	LRCH4	RABL3
LINC00673	PLCD3	PADI2	PIGG	MTHFS

APLP1	RAPGEF5	CALCOCO1	ASUN	BAG2
SOGA3	EMID1	PRCP	KIF23	ATF1
NRG3	SNX32	GDPD5	PRPF8	GTF2I
DLX3	H1F0	MBOAT2	FKBP4	PATZ1
HERC2P10	SPRED3	SLC12A6	PRNP	PARVA
ASTN1	CTSH	RGL3	PNKD	BMI1
SELENBP1	LRG1	INPP5K	SNRPF	ZFAND2B
OASL	KLF8	EMP2	EIF1	CCDC183-AS1
XKR7	TMEM51-AS1	RTN4	DDX10	DNAJC2
FAM47E	TMEM198B	COBL	ARHGAP11A	CHST14
TNFSF12	SESN3	F2RL1	FABP5	TRMT61A
APOBR	NPTXR	CDS2	RIF1	C7orf26
ST8SIA5	NBEA	FBLIM1	MDN1	PSMB10
GJA3	RGS9BP	PALD1	DDX23	SYDE2
GAL3ST4	SMARCA1	GIPC1	UBA2	COL12A1
LINC01132	MT1M	TBL1XR1	SEC14L1	PBRM1
DDX25	DDN	ITPR3	TOP1	NEXN
WDR66	CXorf49	MVB12A	SENP6	ICAM3
HCK	CXorf49B	IGFBP7	GJA9-MYCBP	CTHRC1
BVES-AS1	CXCR3	SH3GLB2	GTF3C2	BAZ1A
TRPM8	GDPD1	PPIP5K1	TCEB3	RNPEPL1
MYZAP	CAMK2B	MANSC1	DHX33	ENOX2
AP3B2	DLX2	ZNFX1	FIGN	IL1R2
ABCB4	DPYSL2	PADI1	PUF60	DKK1
MMP16	TIAF1	JUNB	MKX	HYPK
TUBBP5	NEAT1	FAM168A	DDX21	SNORD20
KIAA0408	MT1F	STARD10	PES1	SNORA75
CPEB1	TGFB1	TRIM38	BCAS2	FNTB
C1R	SLC2A12	STK10	HJURP	CLPX
LINC01271	LINC00891	NFIC	WDR36	MZF1
LOC101927934	LOC100132741	PIK3R3	CAMSAP1	THNSL1
SMARCD3	TBKBP1	SOCS2	RNF214	ERCC6-PGBD3
ZDHHC19	SOWAHA	NFE2L3	EVA1A	HNRNPAB
LOC100128505	DEPTOR	GRHL1	TPD52L2	LOC101926943
SCNN1D	SEZ6L2	KANK2	NOL11	SPRYD3
DYRK3	FOLR1	CHKA	FOXM1	C14orf169
GPR114	PNMAL1	SPEG	ACAT2	OPN3
PLIN5	SHISA2	CORO2A	SMARCD1	TOR3A
SLC8A1	FBXO10	CADM1	MYCBP	ANKRD39
SPOCD1	CFD	MTUS1	CKS1B	LYAR
TP63	PLEKHO1	ITGA3	PA2G4	SFRP1
TLR9	ZFP90	FBXL18	POLR1B	RPL23AP7
CCM2L	HSH2D	TSPYL4	CDC45	TEN1

GNA15	ADORA2A	ZBTB4	MLX	MMAB
S1PR1	TNFSF9	TNFRSF21	AKT1	NCL
ESRRG	NLGN2	GINM1	BCL7B	RNMTL1
STK32A	LINC01137	SH3BGRL2	SRSF4	MESDC1
PLA2G10	C17orf103	RPS6KA1	ZNF721	L3MBTL2
PDLIM4	PLCH1	SERPINB5	DCUN1D5	BEND3
CSPG4	RBPMS2	CTIF	AHSA1	CCNE1
UNC13A	IL10RB	ATP13A2	RTEL1-TNFRSF6B	ZNF41
BMF	MARCH2	CD109	RNF6	ZFX
PRRT3	MYL9	FHL2	SMEK1	ABCA11P
PIK3C2B	FGF13	TRAK1	ZC3H14	C1orf186
FEZF1-AS1	FAM43A	PLCB3	TRMT1	MOCOS
ZNF521	CCDC113	PTRF	ESRRA	CCDC97
TMEM211	ANGPTL3	ALDH2	DIMT1	PPAP2B
MIR3189	STXBP6	IL18	MTPAP	FAM216A
IDH1-AS1	FGFR1	HIBADH	KIAA0368	RPARP-AS1
FAM212B-AS1	NICN1	STIM1	AP1G2	PRMT3
LRRC38	LEMD1	TACC1	DNAJC11	TCHP
C3	KREMEN2	PABPC1L	ATP6V1D	MSTO2P
NCAM1	STYK1	SORT1	DKC1	LTBP2
B4GALNT1	DNAJC18	WWC1	C17orf85	NRDE2
FAM107A	FBLN2	RHBDF2	SMC1A	LINC01127
ZEB1	FMN1	TMEM9	ARID1B	IGFBP5
ZBTB8B	GSN-AS1	FAM102A	MTHFD1L	LOC101927884
MRAP2	SGTB	GLCE	KIAA0020	MBNL1-AS1
FBLL1	TMEM8B	IGFBP2	PREB	NETO2
WDR31	SERPINF2	MOB3A	UBXN4	NFYC
FLJ42351	LYPD6	FADS2	CHMP3	ZNF678
FAM166B	GPSM1	PAM	FNTA	ERCC6
LOC100130452	JAK3	IFNGR2	LSM14A	LOC101927746
PDK4	NEBL	KIAA1522	MAML1	METTL1
RLTPR	ACRC	CHEK2	RPL26L1	HOXA9
MIR6784	ERC2	BCL9	KIAA0754	WBSCR16
SNAI3	MYH14	SDC3	DDX18	NMI
IL1B	USP2	CD24	ZNF740	HMMR
REN	NAT14	RAB25	MPHOSPH10	SH2D4A
LOXL2	UBTD1	TRAFD1	DBF4	RRP1
MEF2C	SLC4A3	TOR1AIP2	FCHO2	HOXA10-HOXA9
SORBS1	GATS	AFF1	TMEM87A	KIAA1875
LOC440461	ORAI2	PBXIP1	TNRC18	SERPINB8
ASPHD1	CACNB3	B2M	BRIX1	LY6K
SAMD9L	PLIN3	CARHSP1	QKI	FAM174B
GXYLT2	CYP2J2	LGMN	PABPC4	ARMC5

RAB42	RIPK3	DBNDD1	FAM3A	RRS1
EEF1DP3	ADAM22	FSCN1	SIPA1L1	POLR3D
APBB1IP	SNTA1	CEP164	HMGB1	PCBD2
ACPP	CACNG4	SERTAD2	HDGF	COA6
NIPSNAP3B	RNF157	IDS	CHTOP	KCNIP3
KLHL30	BCAM	CTDSP2	TRIP13	DSCAML1
CGNL1	PEX11A	RABAC1	USP24	FAM181B
NLGN1	RELL2	PRSS8	PLK1	NSMAF
SEMA6C	CRYL1	ZNF358	PRKCZ	C1orf123
GRID2IP	DMGDH	PRAME	ALMS1	HMMR-AS1
GPR153	ZNF382	TRIB3	GTF3C5	FAM217B
NEFL	TTC39B	MYO1C	PMPCA	ODC1
PIK3R5	SELM	LAMP2	GNG5	TMTC4
CYP2S1	CDRT4	ANO6	NOP14	DUSP4
F2R	FAM47E-STBD1	CLDN3	RTEL1	CEBPD
ACAP1	FREM2	MAGED1	NUCKS1	LRRC56
ELMOD1	AAMDC	SORL1	STAG3L5P-PVRIG2P-PILRB	JAG1
PVRL4	LCP1	ACSL3	CCNB1	ZNF407
AS3MT	PHLDB1	AKAP13	WDR74	FAM86DP
IRAK3	TBC1D10A	XPR1	CDC16	LOC100129518
FLT4	PRSS53	NUSAP1	WIZ	WASH1
BIK	BBS9	RIOK3	TXNDC5	CCNO
SLC16A12	TMEM180	UBE2H	RBM22	CECR5
ADAMTS7	FRMD6	VASP	THEM6	C1orf131
SALL4	C1orf210	CACUL1	SLC29A2	MSTO1
WFDC3	DTX1	POR	PAN3	NIPSNAP3A
KITLG	SMIM8	CDCA7L	PPAN	PDIK1L
FAM13C	STARD9	ETS1	STRIP1	PMS2P9
SGPP2	PAPLN	UGCG	GSK3A	FOXG1
CKMT1A	FGF5	CGN	GPATCH4	HOXA10-AS
GRIN1	KIAA0513	ECE1	TSR1	SIM2
IFIT1	PECR	CASC4	BRCA2	PTGER3
MAF	PAPSS2	CLSTN1	BCLAF1	STS
MLLT4-AS1	MYBPC2	SGMS1	ITPK1	CHCHD4
RTCA-AS1	CAMK1D	KIAA0195	MRPL15	ZIC4
RHBDL1	ATP7B	KRT7	SSH1	SNORD97
HTR1D	LOC284454	MYH9	MRPS30	SNORD82
GFI1	FAM161A	SREBF1	EIF5	LOC100289580
DNAJC22	KIF7	MRI1	EXOSC8	RAB40C
PGAM2	HS1BP3	ANKRD1	MAML2	EDNRA
RASSF2	CCDC80	PVRL2	NUP50	THAP7
RRAD	PCSK5	TNIK	NCLN	PCDH7

INPP5J	AMT	GCA	RINT1	TLX3
TMEM71	ATF3	ACSL1	QTRTD1	HOXA10
LOC100132891	TYRO3	TSC22D4	NXF1	MYL5
LOC101927043	PPARG	SPATS2	RNF44	SOX2
PLCL1	SLC41A2	GNAI2	FUS	RPE65
TMC7	CSF1	SPNS2	TMBIM4	CBX8
AMPH	LOC101926996	SNX2	CASC3	PSEN2
ZNF821	TGM2	PTK7	JADE2	ADAMTS18
C1QTNF2	SYTL4	VHL	FH	DOK7
PELI2	TMEM241	EID1	UFC1	ID4
SEMA4G	FAM229B	PITPNM1	DGKH	TBX18
IL7	FBXL2	MAPK8IP3	YARS2	TTLL12
WNT6	DAB2	ARNTL2	GTF3C6	VPS37C
MORN3	DNAH17	MAP1S	CEBPG	L3HYPDH
TRIM34	LOC730101	MICU1	MFN2	FAM178A
SHF	BAIAP2-AS1	NOTCH2	SEMA3C	SIM1
CCDC148	OGDHL	KIFC2	PSPH	METAP1D
OTUB2	GSTM4	F11R	CALB1	SNORA8
KCNK15	TNFRSF11B	SDCBP	CUL4A	ROR1-AS1
GSTM3	GRB7	UBAP1	SOS1	MLYCD
C12orf80	CHRNB1	CD59	DHX34	ATXN7L2
DMBX1	CMBL	ABCC1	TUBGCP2	SERPINE2
MAPK11	BTN3A1	DCTPP1	FAM98A	GBE1
ZNF221	DUSP10	LRBA	C17orf49	NETO1
GAD1	GNG12-AS1	VCL	POLG	SAYSD1
COL25A1	CD97	LAPTM4B	TCOF1	ABHD16B
ZNF774	GAS6-AS2	ATP8B1	HSPD1	ZNF469
NPBWR1	FHAD1	PHACTR4	LNPEP	WNT3
RNF122	IQCE	PLXND1	CLASRP	RABGGTA
SOX3	SEC14L2	PPP1CB	EMG1	HIST2H2BC
ILDR2	HDAC5	KRT8	RBM39	TUSC1
TSPAN2	MFAP3L	FKBP8	TRMT2A	PLD6
C8orf4	GATSL2	AMFR	ALYREF	FAM53A
HIF3A	SIDT2	BST2	IFT172	EPHX4
RHBDL2	CRIP2	MYO10	SMEK2	DOCK8
SEC31B	HTR7P1	PPFIBP1	VAC14	MFAP5
ATRNL1	LY75	MAGT1	TRPM2	PDE9A
LRRC6	IPO5P1	TIPARP	EXOSC3	PCSK6
TCTN2	IGFN1	FAM129B	SUMO3	LOC101926987
TRANK1	FBXO17	PPP3CA	DDX19A	AVEN
DFNB31	AOX1	BMPR2	SAFB	CHDH
RUNDC3A	TFEB	DFFA	CCDC43	CCDC146
BCL3	FRY	OSBPL9	METTL13	SLC2A9

PLSCR4	TCF7	UBB	TET3	NCOA5
PNMA2	MME	PTK2	DPH7	WNT5B
FOXD3	SPAG4	LASP1	TTC27	AFG3L1P
LIFR	ANK2	KRT18	LYRM4	CCNA1
PON3	PXK	HDAC3	GPR125	ADPRH
CHST6	MFI2-AS1	MKNK2	LDHA	LENG1
SERPINE1	PARP14	MFN1	NCOA6	KPTN
CPNE8	PRX	KIF1B	CDC25A	EPC1
PIK3IP1	PIK3CD	MAST2	CCNA2	SLC26A1
PROM2	CKB	HK1	POP4	RGS7
PRSS22	ABCB9	ACTB	GRPEL1	ZBTB37
PMP22	ZNF514	LAMC1	LSM7	PRDM5
HEYL	ACBD7	PLD3	PPP1R8	FZD8
FER1L4	MAP3K12	PCMT1	DHX37	DNLZ
TLCD2	LIMD2	RFC1	PHF14	NTM
HES2	TCEA2	FYTTD1	ACTR1B	CAP2
LINC01270	TSPAN5	ASAP2	PPIF	PALMD
TMEM74B	FBLN1	SND1	DTYMK	C8orf82
GOLGA6L9	SGK223	SEC63	HSPH1	MCM9
LRRIQ1	REEP1	MAL	SEH1L	MIR6775
ADAMTS7P1	NINJ1	PXN	CTU2	ADAMTS14
VCAN	TNNC1	CMTM6	YRDC	ELFN1-AS1
UBE2Q2P2	FBXO41	CD47	HYAL2	AMPD3
CILP	FAM105A	RHOC	RAB21	GNA14
SPATA6L	KIAA0930	SRPR	MBNL3	FAM201A
ANKRD22	LIPA	CCT2	GEMIN5	ANKMY1
GABRP	SNX29	PSMB2	HGSNAT	NIPAL2
TLR6	XDH	IPO5	DVL1	IRF7
SLC43A1	TMEM44	G3BP1	MYO19	ID2
PLCD1	BTN2A2	KHSRP	SUPV3L1	MRM1
WSCD1	NOL4L	ILF3	IMP4	FAIM
GBP2	STBD1	SLC25A5	POLE3	DUSP2
ACSS3	TMEM132A	GNL3	ARID1A	IL20RA
EYA2	E2F2	ARHGDIA	SLC12A2	PP14571
ABCC6	CEP70	EIF3B	SLC35C1	C19orf71
MLLT3	ARHGAP44	RALGPS2	SPAG7	EME2
TRAM1L1	TRIM14	TRIM28	MAGOHB	FLRT2
LINC01096	AP4S1	PAK2	NCOA2	SMPDL3A
CDC42EP5	LRRC1	BOP1	STK35	MIR6847
LGR6	ARMCX5-GPRASP2	PSMF1	PEX5	MIR6763
LOC727751	MATN2	FAM83H	ZFP91-CNTF	NHEJ1
WFDC2	FAM214B	PSMC1	FAM208B	MPL

DLX5	ZBED8	CYC1	LSM14B	LINC01111
KLHDC8A	CRIP1	H2AFZ	UTP15	SNORA25
CLIC3	MALL	ZNF207	NRG1	ZNF285
SPTB	GPX3	SRSF1	SPAG1	LOC100499489
LOC645638	NOXA1	AURKAIP1	TOMM22	ATP6V0D2
MAP2	CEP68	POLR2H	SLC25A22	CRISPLD2
TMEM159	PTPRU	WDR3	PIGU	MIR4800
PDE4C	L3MBTL1	DDX27	MBNL1	C9orf172
BARHL2	SLC25A42	SERBP1	RBM8A	ASB9
LIMS2	CMTM8	NT5E	ZFHX4	TNRC6C
LOC80154	CPM	SBF1	ZGPAT	CCDC74B
RIPPLY3	EFNA1	PGM1	ASXL2	LIPT2
NRIP3	EPHX1	TIMM50	LOC100288181	ACOT1
HCAR1	BCL6	SRP72	ABLIM1	LIX1
STAG3	TSPAN9	RAN	CSRP2BP	MIR133A1HG
TMEM169	SLC1A3	DNAJC9	CDH13	CMPK2
FGF2	LRRC61	USP5	ASH2L	SLC22A3
C19orf83	LMBR1L	FUBP1	USP36	UNC5CL
MIR4489	FCHSD1	SNX19	NIN	ADAM12
SYT14	CCDC24	CCND2	DUSP6	THAP7-AS1
ACTBL2	TPST2	CDT1	ESF1	KCNJ2
ADAM8	RRAS	GTF3A	POLR2F	LOC101928417
LUCAT1	GCNT2	PGD	CCDC59	KLF15
GNAO1	CDKN2AIPNL	C7orf55-LUC7L2	TNPO1	KDM8
LOC100653515	B4GALNT3	SPIN1	ANP32B	MED15P9
PRSS54	PRKCG	TMEM109	CHRAC1	EDN2
GATSL3	ETNK2	TWF1	SLC25A38	S100G
SFXN3	EVI5L	TIMELESS	CPSF7	MIR302D
HCN3	FMNL3	TFAM	MRTO4	MIR302C
C2orf81	CAMK2D	PSMC4	PAN2	RYR3
MIR6777	SLC9A2	SMPD4	SBDSP1	MIR302B
NRTN	C4orf33	SPOCK2	ARAF	MIR302A
EHD3	EPHB2	DAZAP1	PAF1	ZFHX4-AS1
PLCB1	RNF215	AUP1	ICT1	HNRNPA1P33
GOLGA6L10	ARHGEF3	SEC61A1	NAT10	MIR4706
RPS6KA6	MGAT4A	UBE2Q1	ZFP91	CLYBL-AS1
RPS17	CCBL1	DDX49	SBDS	LINC01125
GALR2	ZFYVE19	PSME3	UCKL1	MIR140
GAL3ST3	SPIRE2	PPP2R1B	RPP40	MKRN3
ELF3	ZSCAN26	BAP1	APRT	KCNJ2-AS1
HOMER2	APBB3	PFKL	TOMM40	CYP4F62P
OOEP	ZFYVE1	PHIP	DCPS	TUBA3E
ANGPTL6	DFNA5	THADA	ELAC2	POTEF

SOD3	GCHFR	SLC39A10	STRADA
ADHFE1	LOC100131564	TBC1D14	ADCK2
GGT6	BLVRB	ERCC3	SMYD5
ACP5	TGFB2	TSC22D1	BUD13
FAM155B	ARSK	KIF20A	RCN1
MAPK8IP1	C10orf55	BIRC5	GMEB2
AGPAT4	CLMN	MRPS2	POM121C
ATP7A	TRERF1	MRPL3	DDRGK1
ACVR1C	C3orf52	USP1	MED27
BVES	LBH	SORD	MARCH1
CLDN9	CREBL2	WDR43	COMMD3-BMI1
RASSF4	PFKFB4	AFG3L2	NOP16
EMP3	SRGAP2	LPHN2	ARRDC3

Supplementary Table 4. Unique genes (total of 638) induced by entinostat in BRCA1-null compared with BRCA1+ ovarian cancer cells (*P*<0.01, t-test, FDR<0.05). Gene expression was measured by RNAseq.

NOVA2	PRR5L	WBP5	ALDH6A1	LINC00092
CXXC4	RAB3IL1	ITGA6	CYP20A1	TUBA3FP
RNY5	TRIM6-TRIM34	HMGN4	GINS4	GP2
CACNA1G	S100A5	UBE2E2	ZACN	DPPA4
ADAMTS10	GRAMD1B	ODF2L	ZFYVE26	LOC100996291
COL2A1	TRIM6	STX10	PDDC1	MS4A7
FAM57B	ESYT3	CDCP1	PQLC2	KANK4
RASGRP2	LURAP1L	CALM2	RIN1	GPR12
RGS11	LOC283070	LYN	NMNAT1	LINC00907
GPM6A	EVA1C	SRC	CAMK2N1	ADRA1A
MYPN	TMEM156	USP54	PANK1	CYP8B1
LOC729966	LPL	BTG3	SLCO2A1	LINC00939
ARHGEF25	EPN3	CLGN	HSCB	HAVCR2
TOX3	GIPC2	CKAP4	UPK3BL	C1orf140
TMC3	FBXO16	ARL6IP6	PHF2	LINC00330
TMEM40	S100A3	DOCK4	CACFD1	SCN2B
FLJ42969	SYT1	B3GNT5	IRGQ	CD82
TDRD5	ZDHHC1	FANCL	ADAT1	RHBG
USHBP1	HIC1	SKAP2	COX18	CR1
TCN1	ZNF883	UBE2W	ZNF76	CALN1
KCNT2	GPR124	VEZT	POLR2J2	TLR10
LINC00635	CACNA1A	MTMR6	MED18	FAM74A4
SDS	MUM1L1	IGF2BP2	RABL2B	LOC101928104
MAP6	UST	UBE2E1	LOC100287098	GDF6
DMRT2	NTF3	SLC43A3	BMS1P4	NAP1L6
OTOGL	ARMCX2	BRE	TSC22D1-AS1	OTX2-AS1
OR1F1	GRIN3B	EFR3A	MDM4	C7
KCND2	TBX6	TTL	DFFB	ITGAX
TMEM63C	SYTL3	TNIP1	WHAMM	SIGLEC8
VAX1	MAMDC2	HN1	RNF125	LOC101927796
SIRPG	PTPLAD2	ELK3	KIF26B	LOC101928401
SV2A	CTB-113P19.1	SRI	SPHK1	SULT2A1
MIR4712	KRTAP2-3	ZNF706	ZNF737	LINC00696
ABCB1	SLC1A1	ABCD3	ILF3-AS1	FENDRR
LY96	MIR1273H	HM13	MAN1B1-AS1	SLC6A14
CLIC2	SULT4A1	MPZL1	PRKCDBP	NDST3
DEGS2	PLVAP	JUN	NME6	POM121L1P
SLC2A14	METTL7B	AMOTL1	UNKL	C8orf31
LOC100192426	KCTD17	AGPS	DYRK1B	

CD163L1	NUDT17	ZMYND8	PPM1K
LOC152225	DEPDC7	TP53BP2	NLRP10
EFHC2	TTC33	REEP3	TRPV1
CLCNKB	FAR2	PKP4	NEXN-AS1
NOTUM	ZNF30	HNRNPUL2	ZNRF3-AS1
FAM19A4	ZNF365	KIAA0100	PERM1
FAM196B	BCHE	NFATC2IP	IKZF3
ADAM28	C16orf98	VAV2	LOC100134868
ETNPPL	TRAF1	KDM2A	SMOC2
GPR4	FAM83A	DCAF11	HHIP
LOC441461	TSPAN33	MROH1	LOC284023
CPNE5	NPR3	INCENP	MIR4751
CCDC173	PARVG	LRFN4	FAM71F2
NRG2	AQP1	CIAO1	CDH4
LDHD	C2orf88	CBX5	LOC101928514
CLEC2B	STEAP1	DGKZ	KIAA1456
LOC101928796	CPLX1	SLC12A7	CCDC171
GPR112	EPB41L4B	ICMT	LINC01105
MIR6794	EFHD1	GTPBP3	CD84
LOC100996385	TMEM200B	TPRG1L	SLC24A4
KRT77	FAM169A	PTPN23	PPP1R26-AS1
KRT1	DENND2A	TENM3	HP09025
AHSG	TP53I11	POM121	RPL23AP64
KRT76	MAP9	FANCA	GPLD1
CDIPT-AS1	PRKAA2	FOXN3	NFAM1
MIR1976	SMIM10	CNTROB	DAND5
SLC16A14	DUSP5	SNHG3	BNIPL
LOC102723641	TMCC3	XAB2	LOC101927415
IL1RN	NPNT	SGPL1	BCL2L15
ARHGEF38	SLC25A45	MAVS	CYP1A2
NIPAL4	PEAR1	MFSD11	C21orf62
FAM133A	FAM101B	ABCD1	LOC100996251
SERP2	TFAP2A	SLC35F6	ARGFX
LOC100505795	C19orf26	LMNB1	PRICKLE2-AS3
BCO2	ALPK3	DHX38	AIPL1
PTGS2	IL23A	MED16	IL12RB1
EBF4	TYMP	TNS3	EMX2OS
CD8A	GPR160	HTT	CCR6
PTGIR	LOX	FURIN	KCNJ11
CD247	LOC100126784	PPP1R16A	SLC5A5
CTTNBP2	F8	ZC3H3	CCL5
AVPR2	KATNAL1	CYHR1	LINC00970
CASP1	NOVA1	PCK2	LINC00574

SLC19A3	DPF1	INSIG1	SIGLEC10
MYO15A	MAP3K7CL	RAB11B	LOC101928738
COPZ2	M1AP	NCAPH2	SPN
TCEAL2	LOC100507373	SQSTM1	FBXL13
CASC15	CYGB	RCE1	MIR4492
FRZB	SYBU	RPS6KA4	FGB
ABCG2	SCEL	B4GALT7	GBP6
WNT10B	SLC7A7	GTSE1	SLC36A2
HNF4G	MRC2	ILVBL	POM121L10P
CSF2	RAB8B	CCNF	C1orf229
DZIP1L	CXCL1	HOOK3	NLRP12
LINGO3	UNC13D	DCXR	LOC101929259
FAM131B	P2RX4	OGFOD3	ADAMTS4
BARX1	FAM131A	NUP43	LOC400644
CXCL2	ABLIM3	LYPD1	SEC14L4
FAXDC2	HOXA3	TMEM192	SGSM1
PDX1	LOC730102	THBS1	LOC284865
ELN	CROCC	PHPT1	FGD5P1
RARRES1	SLC16A7	ZZEF1	VSTM4
ZNF385B	GNG11	CCDC88C	LINC01134
ELMO1	RILPL2	MIB2	ATCAY
ALOXE3	SYS1-DBNDD2	PTPN2	SMIM17
CNTN5	LOC100506071	CRTC1	FLJ30403
TBXAS1	PAQR3	ABCB8	ADAMTSL2
EPB41L3	PRTFDC1	LOC100190986	SRRM2-AS1
GJA4	BCL10	C9orf78	LINC00327
CNTD2	SEMA4B	FBXL6	ABCB5
PILRA	FAM110A	AIF1L	KANTR
PRDM7	CCDC82	ZNF185	C14orf105
ARHGAP24	TNFSF12-TNFSF13	IVD	L1TD1
CCL20	WDR47	C17orf62	LOC101927666
MAGI2	PRSS16	MRPL55	BMS1P5
LRRK2	OSBPL6	ZFYVE21	LOC100506385
CALCRL	TBPL1	SHPK	LUZP2
ST6GALNAC1	ST6GALNAC4	TRIM65	VENTX
RASIP1	PLAGL1	TMPO-AS1	AKR1D1
BATF2	S100A2	SLC1A5	ATP11A-AS1
ADCY4	KLHL5	ASPSCR1	LOC101928580
GCK	BACH1	CWF19L1	LINC01247
LOC100506985	C17orf104	INHBB	TNFSF14
ISM2	ZNF277	GATAD1	PIWIL2
GRIN2D	PLOD2	ORC6	FCAR
TMEM121	HAGLR	VPS53	OR51E2

CEP112	TMEM14A	ZNF33A	ITGAL
VNN1	CLDN11	NADK2	FLJ36777
HAND2	DNAH14	NOM1	LINC01212
ARMC3	TAOK3	POLH	NUTM2B
C11orf70	CLDN12	WDR90	BEND2
DENND6B	ALKBH2	RPP14	LARGE
TLR5	POLR3G	GNL3L	RASGRP4
ESAM	ACAA2	TMEM104	PBOV1
LOC286367	DHX57	TRMT2B	LOC101929224
VAV3	CREB3L2	SLC25A15	UTY
CCDC110	LANCL1	TUBB3	INMT
DLGAP3	IMPA1	IER5L	LOC101929680
TCF24	TMC6	MSH3	LOC101929372
SLC9A5	ZNF219	MEF2BNB	CXorf36
FAM179A	FAM189B	C1orf174	MTHFD2P1
RNF157-AS1	HDGFRP3	TOP3B	MIR1204
ADAM23	PFDN1	PINK1-AS	TAT
NDRG2	ARHGAP12	FAM84B	LOC101926897
NAALAD2	CFL2	HEATR3	DNAJC27-AS1
SKAP1	AIG1	LINC01128	LOC100144595
HTR7	FOSL1	ARSD	RFPL1S
ANXA9	PVT1	EXPH5	LOC283731
TCF7L1	TGFA	PTRHD1	WT1-AS
IL18R1	CEP55	PTPRG	PABPC1P2
TSHZ3	RREB1	FAM83H-AS1	FRY-AS1

Supplementary Table 5. Common genes (total of 1274) induced by entinostat in both BRCA1-null and BRCA1+ ovarian cancer cells (*P*<0.01, t-test, FDR<0.05). Gene expression was measured by RNAseq.

PRTN3	WIPF3	MLF1	CD68	TNFRSF12A
SLITRK3	CDKN1A	CHST2	SLC44A3	AKT1S1
SEMA6B	ARL10	PPM1M	SEC24D	PI4K2B
TESC	NPW	PKIA	MCU	SEMA4C
LPPR3	COL9A3	RFX2	ZHX1	RRP9
PPP2R2B	CCDC88B	HSD11B1L	FAM114A1	PCMTD2
ITGA2B	GDF15	CEACAM19	SPATA20	RAVER1
PALM3	HSD17B6	CCDC88A	RNF212	CDK12
SLC45A1	PPP1R14A	GPR63	SLC39A14	PRR12
SRCIN1	MSRB3	LOC101927051	CD9	DSP
SERPING1	FLJ44313	BLVRA	EPB41L1	ZNF512B
TUBB4A	LEPREL2	PRSS23	SLC25A12	KDELC2
PPARGC1A	EBI3	TP53INP2	PALM2-AKAP2	FGFRL1
DACT3	MAST1	PTX3	AKAP2	CTBP1-AS
KIF5C	SLITRK4	LRP1	CPNE7	FBRSL1
GIPC3	SCAMP5	TLE2	P4HA1	C1orf122
KCNJ8	SNAI1	ZNF215	TENC1	STC2
CCDC151	CYP27B1	KIAA1211	PDE4DIP	RHOT2
PIANP	TRPV4	LAMA1	CERCAM	SSNA1
SOX18	GAMT	PAX9	VPS37B	IGFBP3
NKX6-1	FAM171B	KCTD14	CAPG	IPO4
HMGCLL1	PCDHB17	ZNF211	NEK9	STRA13
SH2D3C	CORO1A	GYLTL1B	TNFSF13	CCND1
HPCAL4	CCDC114	SLC46A3	ARRB2	KAT7
FAM131C	SALL2	PTAFR	FADS3	TPRN
SEPT5-GP1BB	COL1A1	RAB3A	ECI2	DUS1L
RORB	PROCR	DHRS7	LDLRAD2	EP300
SLFN11	LCK	SERPINB7	NIPSNAP1	EIF4G2
C1QL1	TIE1	RAC2	MMP15	DIS3L
COL8A2	NRK	CGREF1	ATP6V0A1	MCRS1
C8orf88	SLC16A2	SERPINB1	TNC	WDR4
SPX	MFAP2	HID1	TICAM1	SIRT1
EXOC3L4	ANKRD29	FAM92A1	FUCA1	HMGN2
CRABP2	LEF1	TPST1	LAYN	SH3PXD2A
GYG2	GDPD3	ST6GALNAC3	MERTK	CTCF
ATP2A3	NBPF14	FGF11	MEX3A	TOP1MT
PRRT4	VWA5A	MEIOB	ECH1	FASN
IGSF10	SYNE1	SDC4	RITA1	SRM
FXYD6-FXYD2	GPRIN2	STX2	ZMYM1	ACTR8

TSLP	FAM167B	SUN3	ARHGEF19	CEP170B
LINC01305	IGFL1	CCDC28B	B3GNT3	HAUS7
SYT9	ABHD6	C3orf55	MAPK3	ALS2CL
NKX2-1	APOE	TSPAN1	EHD2	MAN2A2
ELOVL3	NYNRIN	ABHD8	ABCA3	ANGEL1
ATP8B2	MMP11	CYP2U1	S100A1	CDCA3
TNNT1	CLIP2	RARG	SYTL2	U2AF2
NYAP1	PLEKHA4	TSHZ2	DHX40	PKD1P1
EEF1A2	KIF12	CERK	P2RX5	CTNS
MSI1	PARM1	FAM102B	RBP7	HMGB3
VAT1L	CNGB1	EXTL2	TSPAN15	ULK3
SUSD3	HES7	SAT1	ZNF512	SIX2
SHISA8	MMP17	FAM69B	ADD3	PKP3
CD70	PIP5KL1	KCNK6	CCNYL1	NOL9
BAI2	MAOA	PLA2G16	TACSTD2	CDK10
SEPT3	CILP2	GABARAPL1	CYSTM1	TROAP
PCDH10	DTX4	ZNF697	NUMBL	SEPHS1
COLEC12	RNF128	HCFC1R1	LAMB2	CENPN
ANXA8L1	SNX10	KIAA1377	RAB29	ATP6V0A2
QPCT	GSTA4	DYNC2H1	CD276	DGKQ
NLGN4X	MYRF	ABCC3	GSN	KMT2D
MGAT3	PLEKHH2	RCAN3	BAK1	HYLS1
CYTH4	SNAP91	TMEM67	PERP	PRMT2
RPRM	RAB31	GLDC	IL1RAP	EXOC7
FAM134B	CORO6	COL7A1	HLTF	ARFGAP2
ANPEP	DCHS2	ELOVL7	NME7	RASSF7
LIPH	APBB1	CHN1	HMHA1	LRP8
NECAB1	L3MBTL3	RGL1	GLIS2	CLSPN
ASXL3	CNRIP1	CELSR3	CTTNBP2NL	VPS33A
PALM	ECHDC3	FAM184A	UACA	BRD9
LRCH2	STOM	IFI30	TWSG1	SLC7A5
RNF182	FBXO44	SLC35G2	LTA4H	ZXDC
PDLIM3	TTC39A	MPP1	PCNXL2	DPP9
TRPV2	ENTPD8	IKBIP	MYO18A	TSNARE1
RAB39B	TM6SF1	LARP6	DNASE1L1	BRD1
CELF5	FGFR4	POPDC3	OAZ2	BRMS1
MLXIPL	RASSF5	CDH24	ERGIC1	SRCAP
NCCRP1	ANKRD34A	CHPT1	VANGL2	TMED1
NOTCH3	C11orf45	TRNP1	PDP1	PEF1
EMILIN2	STEAP2	PHF21A	ERRFI1	DOLPP1
SCN9A	PLA2G4A	C1orf115	IFT46	MSL3
MFRP	C15orf48	RGS10	ADA	RIPK1
C1QTNF5	CDH5	EML2	SH3PXD2B	CRAMP1L

FAM153B	IL1A	SYDE1	TMEM25	FEM1A
CHRM4	SLC10A4	ZNF467	TPCN1	PBDC1
STON1-GTF2A1L	ADAMTSL4	FAM132B	SUMO1	NOTCH1
SELV	STX1B	CNTNAP1	HSPG2	ATHL1
NES	CX3CL1	MAP7D2	B4GALT5	TLR4
GIPR	THY1	GPR98	ACSF2	PRKAR1B
KRT2	GLS2	DNMT3B	VAT1	P2RX5-TAX1BP3
NACAD	NGEF	MT1A	NCOA3	BRPF3
REEP2	NFATC4	PROS1	MGAT5	LRRK1
LOC113230	MFI2	IGFBP6	ARRB1	NRP2
KRT72	TUBA1A	ST3GAL2	AHNAK2	ANAPC2
KRT73	SEMA3F	ENPP5	GRINA	RRM2
KRT74	SCN1B	RRAS2	RASA1	FAM57A
KRT71	LOC100507002	CDA	TCIRG1	TDP1
PCDH20	TSPAN10	NRSN2	IGSF3	GOLGA3
MAGEL2	YBX2	SH3BP1	C12orf75	FN3KRP
FSD1	NDRG4	CDR2L	PRDX5	NUDT15
LOC101929613	RAMP2	TRIM46	UCP2	GABPA
SRPX2	COL16A1	CERS4	HDAC1	FAM195B
TNFRSF18	RECK	DDX43	ERO1L	C9orf69
ITGA1	ETV1	CBX6	SOAT1	BTBD6
TSPAN7	IFT81	DAB2IP	CLSTN3	KAT2A
FXYD6	CCDC181	BTN3A2	FHL1	BAIAP2
RTN4RL2	ITGA4	DSE	ARAP1	STAG1
SULT2B1	RAPGEF4	KIAA1467	HS2ST1	PHACTR3
SLC40A1	SYN2	DBNDD2	MTA3	SMARCB1
HOXD10	CNTN1	ROBO1	FKBP10	PER1
CYP24A1	C1orf21	GGT7	NIPAL3	SAMD10
LYPD3	RBM11	CBLC	BCKDK	SMG9
SYNGR3	ISG20	ADD2	NFKBIA	CREBBP
KHDRBS2	GRAMD1C	POLK	SLC29A1	TRMU
ABCG4	ZIC3	KIF3C	TIMP2	MPDU1
NPY1R	TMOD2	KIAA0040	HIP1R	ZNF503
CHRFAM7A	SYT17	ALPK1	KDM5B	CNNM3
SYNGR1	GATA2	SIPA1	FOXO1	FERMT1
FAM65C	TMEFF1	PLS1	LGALS1	KAT5
LOC100507387	HSPA2	RGS14	CAPN12	MAZ
KRT13	NLRC5	SGCE	RNF114	ALDH16A1
FBN3	GLIPR2	ERO1LB	CIB1	ARHGAP23
TMEM61	NRGN	PGAP1	SNX9	SKI
PPM1J	COL17A1	ANXA1	RAB13	SPR
ELOVL2	LRFN1	RCOR2	HSPA5	SLC19A1
KCNH2	SERPINI1	SEMA7A	RASAL1	SP8

TUBB2B	RGS9	RNF24	RNF11	HERPUD2
SEMA4A	KIAA1462	LITAF	ITGAV	AMIGO2
PDZD4	WNT2B	CEBPB	TWF2	NOC4L
SAMD14	ACAD11	WLS	NBEAL2	GLUL
CAND2	TTLL7	MMD	RTN3	KLHL21
CPNE4	GDPGP1	ICAM5	CALU	PCNT
SPTSSB	GPNMB	FAM63A	COPB1	HS6ST1
SLC7A8	WBSCR27	LIMA1	USB1	FAM58A
HPD	NMNAT2	HSD17B11	MTCH1	TAF6L
ELOVL4	ATP2C2	C15orf38	LMNA	ZBED2
THSD1	GPR173	SIGLEC15	GAK	TELO2
AIM1L	PDE4A	ITM2C	MYBBP1A	PDE1C
TMC8	SLC35E4	MRAS	BLOC1S5-TXNDC5	PRDM10
TMEM229B	ARL4D	ICAM1	MCM7	ADCY1
C15orf65	RRAGD	LRRC8C	FAM120A	KAT6B
CRIP3	DENND2C	BSPRY	HGS	SAPCD2
CXCL8	NR4A1	SEPT6	CORO1B	KMT2B
RBM24	RAB26	PCYOX1L	SPON1	TP73-AS1
VPS37D	COL9A2	MTSS1	VCP	SUV39H1
SBK2	MAN1A1	LRRN4	PTBP1	CCDC101
ASAP3	SDC1	RIMS2	BPTF	AJUBA
FAM155A	TNS4	TGFB1I1	TRIP6	PLXNA1
SLITRK5	CD274	SMAD3	RPRD2	INTS10
GRAMD2	DLL3	SPSB1	NUP214	TMEM18
BEND4	KANK3	KCNS3	VPS51	MEGF6
SNCA	C19orf66	GDAP1	ABCF2	TADA2B
BCAS4	ENO2	NPHP3-ACAD11	LEPROT	D2HGDH
AATK	GPC2	ELL2	POLR2C	FAM21C
CCDC136	MIR4260	MYO5A	TACC3	FAM21A
SYN1	JAZF1	MICAL1	SRRM2	MBD3
NXPH4	DSC3	MAP4K2	POLR3E	ZNF641
CXorf57	FBXO2	L1CAM	PARP4	PDCL3
SYT11	CYBRD1	STPG1	UTP20	C14orf80
APH1B	ANKRD6	MFSD6	TIMM44	TONSL
LINC00173	NPL	OCEL1	KPNB1	ARRDC1
GPR37	ARTN	SLC16A5	IFRD2	OPA3
CDKL2	MAPRE3	PRPF40B	POLR1A	ADCK3
RTBDN	SMO	VIM	OTUD4	SLC39A4
CIB2	NPR1	TMEM56	NOC2L	ESRP2
GPR85	SLC8B1	MCAM	ZPR1	DUS3L
RIMS3	GAREML	DKK3	MKI67	RBM23
KCNH3	DNAH2	KRT80	PLEC	CYTH2

COL4A6	BASP1	ITGA5	SCRIB	ADAMTS1
GLT8D2	TNFAIP8	IRAK2	MTOR	NR2F2
NPAS1	LRRC49	WDR54	SETD8	SLC35C2
KCNV1	ADAM19	CD302	PIEZO1	MEPCE
BRSK1	SYPL2	SLC45A3	SAP130	DDX51
SHOX2	KIAA1324L	LPHN1	CLPTM1L	PTPRB
FNDC4	CXXC5	PLAUR	PM20D2	FOXC1
NKX2-8	PTPN13	PGF	GALE	LRRC45
RTN2	PAPL	IFT80	C15orf52	C1orf198
LCN10	MED12L	PJA1	TLE1	BARX2
SLC13A5	PBX1	TRIB1	GRB10	SLC52A2
RPRML	TOX2	HIVEP2	ATAD3A	PHF19
PRRT2	SDR16C5	SUFU	FBXW5	TRHDE
SYNPO	FBXL16	SAT2	H2AFX	C17orf70
CA11	ASIC1	POLR3GL	UNG	RABEP2
P2RY6	RCN3	B3GNT2	POLR2A	ITFG2
GRHL3	PLEKHB1	EMP1	CFLAR	ACOX3
SLITRK2	PACSIN1	COCH	HNRNPH1	MXD4
TMEM240	SLC29A4	UGT8	NUP188	SLC6A9
TNFRSF11A	ACOXL	TMEM56-RWDD3	ZBTB44	SLC5A6
HAP1	TRABD2A	CDH1	CPSF2	DNPEP
SEMA3B	KDELR3	EGFL7	LRPAP1	THBD
KLHDC8B	KIAA1161	TNFAIP3	SETD1A	KCTD15
PPM1N	TNFAIP2	LTBP1	MARVELD1	ATF5
HSD17B14	MATN3	USP35	PRKAG1	IBA57
GMPR	PCDHB6	PFKM	ACTG1	LINC00941
ZNF385A	LMO2	SPARC	RBM3	U2AF1L4
RASSF6	PDZD7	PAK1	PFAS	AP5S1
PLB1	KRT15	ST6GALNAC6	EBNA1BP2	DPP3
AMZ1	TMEM45B	DOCK3	OBSCN	TRHDE-AS1
SLC51A	PLXNB3	DOCK6	PTPRK	KMO
PLAT	CAMK2N2	VIM-AS1	NDOR1	N4BP2
MLLT11	BATF3	CYFIP2	CAD	CDAN1
TMCC2	NXPE3	CYR61	FAM166A	AJAP1
RBM20	MPP2	LEPREL1	PROSER1	HCFC1
TRPA1	COL4A5	PAIP2B	PCGF3	PCDH19
FYN	SNPH	PCOLCE	RBM14	PIGW
A4GALT	CCDC126	TC2N	WDR5	GUCD1
NAP1L5	HBEGF	ZNF217	POLDIP3	ADI1
PLA2R1	HOXD8	ARNTL	UBTF	TAX1BP3
OLFML2A	LAMB3	MARCH9	PHRF1	MARVELD3
CD14	HMOX1	SH2B3	MISP	LRRN1
TRPC1	MTMR11	DZIP1	RNH1	C2orf54

NXPH3	ARHGEF10L	ZP3	USF2	ATP10A
MAPK15	PAQR8	ATP2B4	SDCCAG3	LANCL2
PLEKHG6	TMEM255A	B4GALT1	SURF2	PLEKHN1
UNC5D	SGCB	NME4	TTF2	RGS12
LHX9	FRRS1	DIXDC1	UPF1	ING5
PLAC1	KCNN4	PHLDB2	TCF25	CDH6
LINGO2	THEMIS2	C1GALT1	GNA11	FOXQ1
LAT2	HPSE	TMEM55A	PWP2	PER2
IFFO1	STXBP1	PALLD	ARMC6	OSGIN1
G0S2	KLHL13	LY75-CD302	TMEM184A	LOC730183
FBLN5	NAP1L3	ARHGEF40	ADRM1	NOG
PHYHIPL	FKBP1B	MSN	WDR6	PRDM11
KISS1R	AGAP2	CLDN4	ZMYND19	FBXL19-AS1
TJP3	CACNB4	GLO1	TEAD4	ST6GAL2
TMEM45A	BCORL1	ABI2	TMEM115	FAT3
STON1	IRF5	CDH3	CTBP1	CA2
NSG1	LOC729970	DNAJC25-GNG10	RECQL4	ADAMTS15
CCR10	FCHO1	GDF11	PRKRA	TMEM132B
SECTM1	RBPMS	PKIG	LRRC47	EDAR
ADAP2	LMTK3	PSIP1	TMEM43	LOC101929698
GABRB2	RAB15	HIP1	CCDC86	KRT3
CARNS1	MPP7	GNG10	MCM4	KRT4
CPT1C	ANO5	JUP	TYSND1	KRT78
FAM171A2	GDA	LAMC2	MAP2K7	KRT79
PLIN2	EPOR	TM4SF1	GRWD1	
PLIN4	GLRX	SLC27A1	MTR	
SLCO4C1	FKBP7	C15orf39	FLII	
CSDC2	LTBP4	LZTS3	ATAD3B	
EFNB3	MXRA8	KRT19	ZC3H18	
ESPN	AGAP2-AS1	TBC1D9	BDH1	
USP44	FA2H	CSRP1	SMG5	
LGI2	ZNF711	SLC48A1	PRICKLE1	
HOXD11	PRKACB	GPR161	WDR81	
ANXA6	TNFSF10	AKT3	RPUSD1	
IQCD	EML6	SQRDL	GCDH	
RAB6B	ELOVL6	LAMA3	CPSF1	
PPP4R4	NTN4	TBC1D1	CDCA8	
C1QTNF1	ITGB3	PDLIM1	RCC2	
HSPA12A	PYCARD	CLDN1	KEAP1	
EFNA3	SHISA4	ARMCX1	HECTD1	
ASGR1	TTC7B	FAM50A	SPAG5	
ENO3	HKDC1	DLG4	TUBB4B	
TEX15	THRA	DNM1	FAM20B	

VWA1	DEGS1	LINC00998	TMPO
ZNF204P	MAPRE2	CPNE2	ECSIT
UBE2L6	DGKA	VKORC1	C9orf114
S100A14	MOXD1	TMEM263	STEAP3
BEX1	PSD3	TBC1D31	C7orf49
PMEL	CAMK1	RHOB	VPRBP
SRPX	CHCHD10	SHC1	TMEM139

Supplementary Table 6. Ingenuity Pathway Analysis of differentially expressed genes in BRCA1-null compared with BRCA1+ ovarian cancer cells.

**Top Networks**	**Score**	
Cell signaling, nucleic acid metabolism, small molecule biochemistry	36	
Cancer, gastrointestinal disease, organismal injury and abnormalities	33	
Auditory disease, behavior, cardiovascular disease	32	
Embryonic development, tissue development, developmental disorders	31	
Cancer, organismal injury and abnormalities, gastrointestinal disease	31	
**Molecular and Cellular Function**	**Molecules**	***P*-value**
Cellular movement	702	5.32E-10-1.69E-53
Cellular development	995	5.12E-10-4.82E-37
Cellular growth and proliferation	1057	5.12E-10-8.41E-31
Cell morphology	721	5.12E-10-1.70E-30
Cell-to-cell signaling and interactions	588	3.70E-10-4.82E-30
**Top Canonical Pathways**	**Overlap (%)**	***P*-value**
Hepatic fibrosis/Hepatic stellate cell activation	36.1	3.07E-16
Axonal guidance signaling	24.4	7.72E-12
Role of macrophages, fibroblasts and endothelial cells in rheumatoid arthritis	26.7	3.86E-11
Role of osteoblasts, osteoclasts and chondrocytes in rheumatoid arthritis	26.5	2.00E-08
Human embryonic stem cell pluripotency	30.6	3.17E-08
**Top Upstream Regulators**	***P*-value of overlap**	**Predicted Activation**
TNF	2.12E-61	Activated
TGFB1	3.37E-51	Activated
IFGN	1.49E-36	Activated
Beta-estradiol	1.86E-35	
SMARCA4	2.22E-33	Activated

Supplementary Table 7. Ingenuity Pathway Analysis of differentially expressed genes in BRCA1-defficient compared with BRCA1-normal ovarian cancer tumors from the TCGA database.

**Top Networks**	**Score**	
Metabolic Disease, Antigen Presentation, Cell Death and Survival	33	
Auditory Disease, Hereditary Disorder, Neurological Disease	33	
Cancer, Cell-to-Cell Signaling and Interaction, Connective Tissue Disorders	30	
Hereditary Disorder, Ophthalmic Disease, Organismal Injury and Abnormalities	30	
Post-Translational Modification, Cellular Compromise, Cellular Function and Maintenance	30	
**Molecular and Cellular Function**	**Molecules**	***P*-value**
Cellular Growth and Proliferation	975	1.61E-03-2.17E-11
Cell Death and Survival	966	2.61E-03-1.11E-09
Cellular Function and Maintenance	770	2.56E-03-5.10E-07
Cell-to-Cell Signaling and Interaction	324	2.62E-03-9.73E-07
Molecular Transport	574	2.47E-03-1.15E-06
**Top Canonical Pathways**	**Overlap (%)**	***P*-value**
TREM1 Signaling	41.3	6.21E-08
Antigen Presentation Pathway	45.9	1.09E-05
Phagosome Maturation	29.2	9.75E-05
Role of IL-17A in Psoriasis	61.5	1.98E-04
Altered T Cell and B Cell Signaling in Rheumatoid Arthritis	30.7	2.37E-04
**Top Upstream Regulators**		***P*-value of overlap**
HNF4A		7.45E-19
TP53		1.85E-10
TGFB1		1.47E-09
FAS		7.80E-08
ESR1		5.94E-07

Supplementary Table 8. IFN-γ-regulated genes that differed in expression between BRCA1-null compared with BRCA1+ UWB1.289 ovarian cancer cells.

**Gene**[1]	**Gene Expression**[2]	
	**Fold-change**	**Activation status**[3]
EHF	166952.1	Inhibited
**OAS2**	15335509.4	Activated
**IFI44**	178979.6	Activated
**FGF2**	901.4	Activated
**DKK1**	273.7	Activated
**ITGA5**	13947.8	Activated
**CD40**	580.3	Activated
**IL1A**	494.9	Activated
**OAS3**	731.5	Activated
**MUC1**	91.8	Activated
**CASP4**	6.8	Activated
**ALDH1A3**	13.2	Activated
**LAMC2**	128.1	Activated
CAV1	28.1	Inhibited
CDH13	28.1	Inhibited
**IRF9**	30.3	Activated
**APP**	6.7	Activated
**E2F2**	-19.8	Activated
**ADORA1**	-19.2	Activated
TRIL	-63.6	Inhibited
CEBPA	-1051.8	Inhibited
GDF15	-18.3	Inhibited
CD14	-106.5	Inhibited
FBLN1	-105.7	Inhibited
IL10RA	-1590.7	Inhibited
**FLRT2**	-113047.6	Activated
FLT1	-15.0	Inhibited
RUNX3	-348614.5	Inhibited
SERPINA1	-971.7	Inhibited

[1]Activated genes are in bold characters.
[2]Measured by RNAseq.
[3]Determined by IPA.

Supplementary Table 9. Expression (fold-change) of IFN-γ pathway genes measured by RNAseq in BRCA1-null and BRCA1+ UWB1-289 ovarian cancer cells treated or untreated with the HDAC inhibitor entinostat. Only genes that changed in at least one of the comparisons below were included.

Gene	BRCA1-null vs BRCA1+	Entinostat-treated[1] BRCA1+ *vs* BRCA1+	Entinostat-treated[1] BRCA1-null *vs* BRCA1-null
IRF1	-	6.8	-
IRF5	5.12	-	5.46
IRF7	12.759	-5.98	-
IRF9	30.297	-	-
SOCS2	-11.567	2.29	-
IFNGR2	-	2.1	-

[1]0.5 µM entinostat for 24 hours
